# Amamistatins isolated from *Nocardia altamirensis*

**DOI:** 10.3762/bjoc.18.40

**Published:** 2022-03-30

**Authors:** Till Steinmetz, Wolf Hiller, Markus Nett

**Affiliations:** 1 Department of Biochemical and Chemical Engineering, Laboratory of Technical Biology, TU Dortmund University, Emil-Figge-Strasse 66, 44227 Dortmund, Germany; 2 Department of Chemistry and Chemical Biology, TU Dortmund University, Germany

**Keywords:** actinomycete, amamistatin, *Nocardia*, siderophore, structure elucidation

## Abstract

Four new phenolic siderophores were isolated from the actinomycete *Nocardia altamirensis* along with the known natural product amamistatin B and a putative biosynthetic shunt product. The structures of all compounds were elucidated through 1D and 2D NMR analyses as well as mass spectrometry. The iron-chelating properties of the retrieved metabolites were evaluated in a chrome azurol S assay.

## Introduction

Iron is known to easily interconvert between a reduced ferrous (Fe^2+^) and an oxidized ferric state (Fe^3+^). This feature makes iron very useful as an enzyme cofactor for the shuffling of electrons. As a consequence of this, the transition metal is involved in many fundamental biological processes, such as respiration, photosynthesis, or nitrogen fixation [1]. In order to achieve iron homeostasis, organisms must be able to control the uptake of this important nutrient from the environment. In bacteria and fungi this is usually accomplished with the help of siderophores [2]. Siderophores are small molecules which, upon secretion, solubilize and coordinate ferric iron with high affinity. The ability to bind metallic ions depends on the presence of suitable ligand groups. Hydroxamates, 2,3-dihydroxybenzoates, and α-hydroxycarboxylates make excellent bidentate ligand groups, as their negatively charged oxygen atoms can maintain strong interactions with ferric iron [3]. But there are also many siderophores featuring ligand groups with nitrogen or sulfur as donor atoms [4]. The siderophore–iron complexes are recognized by highly selective microbial transporters. Following their translocation into the cell, the bound iron is released via a reductive or hydrolytic mechanism [2]

Members of the genus *Nocardia* are filamentous Actinobacteria, which live as saprophytes and were reported from a variety of environments. Although the soil is likely the primary reservoir of these bacteria, a considerable number of *Nocardia* strains was isolated from human tissue and can be associated with infectious diseases [5]. The genus *Nocardia* as a whole is considered to be a rich source of bioactive secondary metabolites, including many structurally diverse siderophores [6]. Up to now, however, chemical studies in this taxonomic group mainly focused on pathogenic strains. In the present study we explored siderophore production in the bacterium *Nocardia altamirensis* DSM 44997, which had originally been isolated from a microbial cave wall community and represents a non-pathogenic strain [5]. Our analysis led to the identification of six iron-chelating molecules belonging to the amamistatin family. Four of these compounds (**1**–**4**) have not been reported before. The isolated metabolite **5** represents the previously described siderophore amamistatin B [7], while compound **6** was before only known as a decomposition product of a synthetically prepared obafluorin derivative [8].

## Results and Discussion

**Figure 1:**
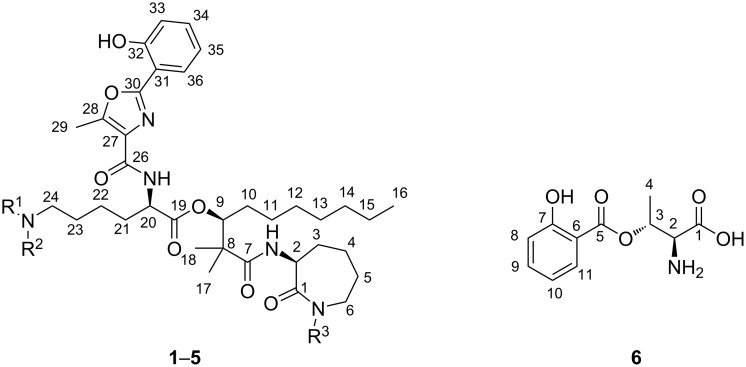
Chemical structures of amamistatins (**1**–**5**) and a putative biosynthetic shunt product (**6**) isolated in this study. **1**: R^1^ = CHO, R^2^ = H, R^3^ = H; **2**: R^1^ = CHO, R^2^ = H, R^3^ = OH; **3**: R^1^ = H, R^2^ = H, R^3^ = H; **4**: R^1^ = H, R^2^ = OH, R^3^ = H; **5**: R^1^ = CHO, R^2^ = OH, R^3^ = OH.

To induce siderophore biosynthesis in *N. altamirensis* DSM 44997, the bacterium was grown in minimal medium without the addition of iron salts. Initial evidence for siderophore production was obtained from a chrome azurol S (CAS) assay [9], which showed a clear color change from blue to pink upon testing of the culture broth. Several batch fermentations of *N. altamirensis* DSM 44997 were then carried out to secure sufficient material of the responsible iron chelator(s) for isolation and structure elucidation. The metabolites secreted into the culture broth were recovered post fermentation with the adsorber resin XAD-7. After removal of the culture supernatant by filtration, the adsorbed compounds were eluted from the resin with methanol. The resulting extract was concentrated to dryness and a first preliminary fractionation was accomplished by flash column chromatography on reversed-phase silica gel using increasing concentrations of methanol in water as eluent. Fractions that showed a color change in the CAS assay were pooled and subjected to semipreparative reversed-phase HPLC. This led to the isolation of six CAS active compounds (**1**–**6**; Figure 1).

The major metabolite **1** (12 mg) was obtained as a slight reddish oil. High resolution (HR) ESIMS measurement revealed a molecular ion at *m/z* 684.3969 [M + H]^+^, which suggests a molecular formula of C_36_H_53_N_5_O_8_ (calcd for C_36_H_54_N_5_O_8_, 684.3972) and corresponds with 13 degrees of unsaturation. An inspection of the ^13^C NMR spectrum confirmed the number of carbon atoms derived from the MS measurement (Table 1).

**Table 1:** ^1^H and ^13^C NMR spectroscopic data for amamistatins (**1**–**4**) in methanol-*d*_4_.

Pos.	**1** (amamistatin C)		**2** (amamistatin D)		**3** (amamistatin E)		**4** (amamistatin F)	

	δ_H_, M (*J* in Hz)	δ_C_	δ_H_, M (*J* in Hz)	δ_C_	δ_H_, M (*J* in Hz)	δ_C_	δ_H_, M (*J* in Hz)	δ_C_

α-amino-ε-caprolactam								

**1**		176.9		170.7		177.1		177.1
**2**	4.46 dd (1.6, 11.3)	53.6	4.54 dd (1.3, 11.6)	52.9	4.51 m	52.0	4.49 m	53.6
**3**	1.45 m, 1.89 m	32.0	1.56 m, 1.92 m	31.8	1.46 m, 1.91 m	32.0	1.48 m, 1.57 m	32.0
**4**	1.90 m, 1.77 m	29.0	1.79 m	28.7	1.92 m, 1.77 m	29.0	1.92 m	29.0
**5**	1.30 m	30.3	1.80 m, 1.62 m	27.2	1.31 m	30.3	1.78 m, 1.29 m	30.3
**6**	3.22 m	42.6	3.93 dd (13.8, 16.0), 3.67 dd (5.0, 16.0)	54.2	3.27 m	42.6	3.28 m, 3.22 m	42.6
								

2,2-dimethyl-3-hydroxydecanoic acid								

**7**		176.5		176.8		176.6		176.5
**8**		47.4		47.5		47.4		47.4
**9**	5.09 dd (2.5, 10.4)	80.3	5.17 dd (2.3, 10.3)	80.3	5.14 dd (2.4, 10.5)	80.5	5.15 dd (2.3, 10.5)	80.5
**10**	1.57 m, 1.49 m	31.0	1.59 m, 1.53 m	31.0	1.58 m, 1.49 m	30.9	1.57 m, 1.49 m	30.9
**11**	1.25 m	27.0	1.31 m	26.9	1.25 m	27.0	1.25 m	27.1
**12**	1.19 m	30.2	1.25 m	30.3	1.21 m	30.2	1.21 m	30.2
**13**	1.28 m	29.8	1.24 m	30.4	1.31 m	29.6	1.31 m	30.3
**14**	1.20 m	32.9	1.24 m	32.9	1.20 m	32.9	1.24 m	32.9
**15**	1.22 m	23.7	1.19 m	23.7	1.22 m	23.7	1.26 m	23.5
**16**	0.82 (6.8)	14.4	0.86 t (6.8)	14.4	0.86 t (6.8)	14.4	0.85 t (6.8)	14.3
**17**	1.15 s	23.7	1.21 s	22.9	1.18 s	23.7	1.18 s	23.7
**18**	1.15 s	21.6	1.21 s	21.7	1.18 s	21.4	1.18 s	21.4
								

lysine								

**19**		172.5		172.8		172.3		172.3
**20**	4.62 dd (5.3, 9.9)	54.2	4.66 dd (5.1, 10.0)	54.0	4.68 m	53.6	4.69 m	53.7
**21**	2.03 m,1.90 m	31.6	2.05 m, 1.97 m	31.6	2.03 m, 1.91 m	31.5	2.09 m, 1.94 m	31.4
**22**	1.58 m, 1.49 m	24.6	1.60 m, 1.51 m	24.5	1.81 m, 1.54 m	24.2	1.55 m	24.1
**23**	1.60 m, 1.76 m	29.7	1.62 m, 1.80 m	29.8	1.94 m	27.6	1.74 m	27.8
**24**	3.22 m	38.6	3.26 m	38.6	3.86 m	64.9	2.96 m	40.5
**25**	8.00 s	163.9	8.03 s	163.9	–	–	–	–
								

asteroidic acid								

**26**		163.5		163.5		163.5		163.6
**27**		129.7		129.8		129.7		129.7
**28**		154.4		154.4		154.5		154.5
**29**	2.69 s	11.7	2.73 s	11.7	2.74 s	11.7	2.72 s	11.7
**30**		159.8		159.8		159.9		159.9
**31**		111.7		111.8		111.7		111.7
**32**		158.1		158.0		158.0		158.0
**33**	7.03 dd (1.2, 8.3)	118.3	7.05 (0.9, 8.5)	118.2	7.06 dd (1.2, 8.4)	118.2	7.06 dd (1.2, 8.4)	118.2
**34**	7.38 ddd (1.7, 7.3, 8.3)	133.9	7.42 (1.6, 7.4, 8.5)	133.9	7.42 ddd (1.8, 7.3, 8.4)	134.0	7.43 ddd (1.7, 7.4, 8.4)	134.0
**35**	6.97 ddd (1.2, 7.3, 7.9)	120.9	7.02 (0.9, 7.4, 7.9)	120.9	7.02 ddd (1.2, 7.3, 8.1)	121.0	7.02 ddd (1.2, 7.4, 8.1)	121.0
**36**	7.83 dd (1.7, 7.9)	127.5	7.87 dd (1.6, 7.9)	127.6	7.87 dd (1.8, 8.1)	127.5	7.87 dd (1.7, 8.1)	127.5

**Figure 2:**
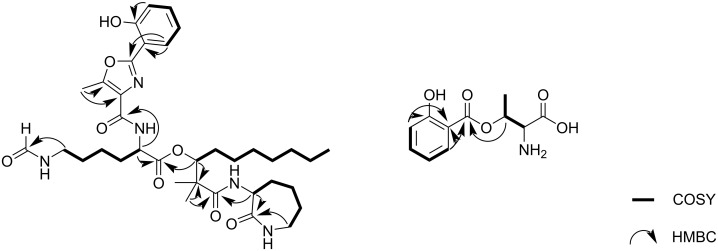
^1^H,^1^H COSY and selected ^1^H,^13^C HMBC correlations in compounds **1** and **6**.

Furthermore, the observed chemical shifts indicated that 14 carbons must be sp^2^-hybridized. Six of them form carbon–heteroatom double bonds (δ_C_ 176.9, 176.5, 172.5, 163.9, 163.5, 159.8 ppm) and eight of them are engaged in carbon–carbon double bonds (δ_C_ 158.1, 154.4, 133.9, 129.7, 127.5, 120.9, 118.3, 111.7 ppm). Therefore, compound **1** must feature three ring structures in order to comply with the required degrees of unsaturation. Proton–proton correlation spectroscopy (COSY) revealed four spin systems, which could be connected through ^1^H,^13^C long-range correlations (Figure 2) determined in a heteronuclear multiple bond correlation (HMBC) experiment. The first spin system comprises the proton signals at δ_H_ 4.46 (dd, *J* = 11.3, 1.6 Hz, H-2), 3.22 (m, H_2_-6), 1.90 (m, H-4a), 1.77 (m, H-4b), 1.89 (m, H-3a), 1.45 (m, H-3b), and 1.30 ppm (m, H_2_-5). HMBC correlations from H-2 and H-6 to the carbonyl carbon C-1 in combination with the observed chemical shifts reinforced the assumption of an α-amino-ε-caprolactam structure. An HMBC correlation from H-2 to a carbonyl carbon at 176.5 ppm (C-7) allowed the linkage of this moiety with a 2,2-dimethyl-3-hydroxydecanoic acid residue, which was identified on the basis of 2D NMR data. In the HMBC spectrum, the singlet methyl protons H_3_-17 and H_3_-18 show correlations to C-7, a quaternary carbon at 47.4 ppm (C-8), and an oxymethine carbon at 80.3 ppm (C-9). COSY and HMBC data established the alkyl chain from CH-9 to CH_3_-16. A correlation between H-9 and C-19 in the HMBC spectrum unveiled the ester linkage with a lysine residue. The spin system of the latter includes proton resonances at δ_H_ 4.62 (dd, *J* = 9.9, 5.3 Hz, H-20), 2.03 ppm (m, H-21a), 1.90 ppm (m, H-21b), 1.58 (m, H-22a), 1.49 (m, H-22b), 1.76 (m, H-23a), 1.60 (m, H-23b), and 3.22 ppm (m, H_2_-24). HMBC correlations indicated the presence of a formyl group at the ε-amino group of this residue. The fourth spin system belongs to a 1,2-disubstituted benzene moiety, featuring proton signals at δ_H_ 7.03 (dd, *J* = 8.3, 1.2 Hz, H-33), 7.38 (ddd, *J* = 8.3, 7.3, 1.7 Hz, H-34), 6.97 (ddd, *J* = 7.9, 7.3, 1.2 Hz, H-35), and 7.83 ppm (dd, *J* = 7.9, 1.7 Hz, H-36). The upfield-shifted resonances of H-33 and H-35 suggested an electron-donating substituent at C-32 and the chemical shift of the latter (δ_C_ 158.1 ppm) supported the assignment of a hydroxy function in this position. From an analysis of the chemical shifts of the remaining carbon atoms (C-26 to C-30) as well as HMBC correlations, we concluded the presence of a 5-methyloxazole-4-carboxylate-derived residue adjacent to the benzene ring. This partial structure is a known motif in some siderophores and is referred to as asteroidic acid [7,10–11]. Due to an HMBC correlation from H-20 to C-26 the asteroidic acid moiety can be connected with the rest of the molecule, thereby completing the determination of the planar structure of **1**.

**Figure 3:**
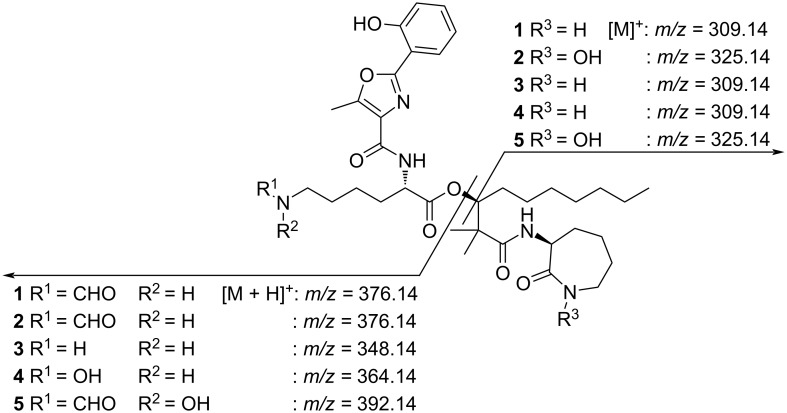
MS–MS fragmentation of amamistatins (**1**–**5**).

Compound **2** (1.1 mg) was obtained as a clear oil. It possesses a molecular ion at *m/z* 700.3921 [M + H]^+^, which is consistent with a molecular formula of C_36_H_53_N_5_O_9_ (calcd for C_36_H_54_N_5_O_9_, 700.3922). The difference of 16 mass units in comparison to compound **1** suggests the presence of an additional oxygen atom. Comparison of the NMR data with **1** reveals the most significant chemical shift deviations in the α-amino-ε-caprolactam moiety. Since the splitting pattern of the corresponding proton signals is consistent with **1**, the lactam nitrogen must be hydroxylated in **2**. Tandem MS data is consistent with this assumption (Figure 3).

Compound **3** (1.8 mg) was isolated as a clear oil. Its molecular formula was established as C_35_H_53_N_5_O_7_ by the [M + H]^+^ ion at *m/z* 656.4019 (calcd for C_35_H_54_N_5_O_7_, 656.4023). Analysis of 1D and 2D NMR spectra confirmed that the aldehyde group in position 25 is missing. Therefore, compound **3** represents the *N*-desformyl analogue of **1**.

Compound **4** (1.9 mg) was obtained as a reddish oil. Its molecular formula of C_35_H_53_N_5_O_8_ was determined by HRESIMS with a molecular ion at *m/z* 672.3966 [M + H]^+^ (calcd for C_35_H_54_N_5_O_9_, 672.3972). Similar to **3**, compound **4** is also missing a formyl group at the ε-amino group of its lysine residue. However, its increased mass of 16 Da indicates the presence of an additional oxygen atom. A comparison of the NMR data with **3** revealed that the chemical shifts of CH_2_-24 are significantly shifted upfield. In contrast, all other resonances are highly conserved and no difference in the splitting pattern was observed. This suggests the presence of an electron-donating hydroxy group next to the ε-amino group in the lysine moiety. The assumption of a hydroxylamine function is consistent with tandem MS data, which showed mass shifts of 16 Da in the corresponding region in comparison to compound **3**.

Compound **5** (0.7 mg) was obtained as a clear oil. Its molecular formula of C_36_H_53_N_5_O_10_ was determined by HRESIMS with a molecular ion at *m/z* 716.3820 [M + H]^+^ (calcd for C_36_H_54_N_5_O_10_, 716.3820). A comparison with published data led to the identification of compound **5** as the known siderophore amamistatin B [7].

Compound **6** (2.4 mg) was isolated as a slight reddish oil. The molecular formula of C_11_H_13_NO_5_ was derived from a molecular ion at *m/z* 240.0890 [M + H]^+^ (calcd for C_11_H_14_NO_5_, 240.0877). An inspection of the ^13^C NMR spectrum confirmed the calculated number of carbon atoms (Table 2). Furthermore, a total of eight sp^2^-hybridized carbons can be assigned based on their chemical shifts. Two of them are engaged in carbon–heteroatom double bonds (δ_C_ 170.0, 169.3 ppm) and six of them form carbon–carbon double bonds (δ_C_ 163.0, 137.5, 131.5, 120.5. 118.5, 113.0 ppm). This indicates the presence of one ring structure to comply with the required six degrees of unsaturation. With the help of COSY and HMBC data two residues were identified, including a threonine and a salicylic acid moiety. The two partial structures can be connected via an ester bond due to a key HMBC correlation from H-3 to C-5.

**Table 2:** ^1^H and ^13^C NMR spectroscopic data of isolated compound **6** in methanol-*d*_4_.

Position	Compound **6**	
	
	δC	δH, M (*J* in Hz)

threonine		

**1**	169.3	
**2**	57.7	4.38 d (3.6 Hz)
**3**	70.6	5.78 dq (3.6, 6.7 Hz)
**4**	16.9	1.57 d (6.7 Hz)

salicylic acid		

**5**	170.0	
**6**	163.0	
**7**	113.0	
**8**	118.5	6.97 dd (1.0, 8.4 Hz)
**9**	120.5	6.93 ddd (1.6, 7.2, 8.4 Hz)
**10**	137.5	7.53 ddd (1.0, 7.2, 8.0 Hz)
**11**	131.5	7.98 dd (1.6, 8.0 Hz)

To analyze the configuration of the isolated compounds, their optical rotations were measured and compared with literature values of known natural products. The optical rotation of compound **5** was consistent with the reported value for amamistatin B (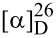 = −9.8) [7]. Similar optical rotations were determined for **1**, **3**, and **4** (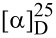 = −1.4, −11.8, and −11.5, respectively). Furthermore, these compounds did not show any discrepancies in their NOESY spectra in comparison to **5**. This suggests that **1**, **3**, **4**, and **5** possess the same absolute configuration. Measurements of **2** were not possible due to the low quantity of isolated material, but it stands to reason that its configuration is identical to **5** under the assumption of a shared biosynthetic origin. Compound **6** shows an optical rotation of 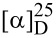 = −10.6, which is consistent with the published value of pseudomonin A (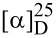 = −9.5) [12]. This natural product is structurally closely related to **6**, featuring a terminal amide instead of a carboxylic acid function. Due to the similarity of both molecules, we propose that compound **6** possesses the same absolute configuration as pseudomonin A.

The affinities of compound **1**–**6** for the coordination of ferric iron were evaluated with the CAS assay [9] and compared with EDTA [13]. While the half-maximal displacement concentration (DC_50_) value of **5** (198 ± 10 μM) was comparable with EDTA (173 ± 6 μM), the other tested amamistatins were much weaker chelating agents (**1**, 3359 ± 181 μM; **2**, 1074 ± 65 μM; **3**, 3268 ± 183 μM; **4**, 3298 ± 261 μM). The increase of the DC_50_ values (**5** < **2** < **1** ≈ **3** ≈ **4**) was in full accordance with the structural features of these molecules. Amamistatin B (**5**) exhibits three bidentate ligand groups and can thus form an octahedral complex with ferric iron. The removal of one of these groups (e.g., the hydroxamate function at the ε terminus of the lysine residue) already leads to a considerable loss of iron affinity, as observed for compound **2**. This trend continues in **1**, **3**, and **4**, each of which features only a single bidentate ligand group. Compound **6** possesses the least iron affinity with a DC_50_ value of 5008 ± 222 μM. The results show a clear correlation between the number of ligands in a molecule and its iron affinity.

**Figure 4:**
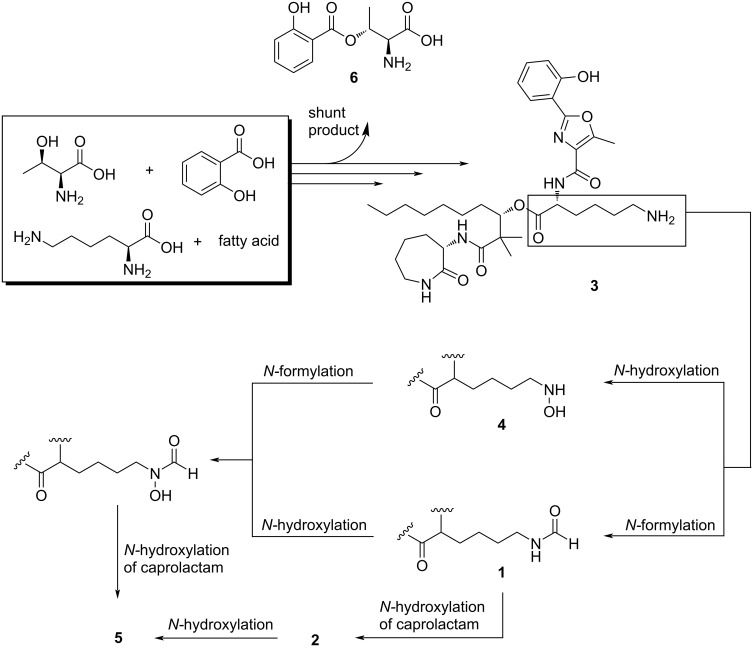
Proposed biosynthesis of amamistatins isolated in this study.

On the basis of biosynthetic precedence [14], a plausible pathway for the natural products isolated in this study can be postulated (Figure 4). According to this proposal, compounds **1**–**4** are intermediates in the formation of **5**, which has the strongest iron affinity and likely represents the end product of the pathway in *N. altamirensis* DSM 44997. Previous studies have demonstrated that, in nature, hydroxamates arise from the oxidation of terminal amino groups in amino acid side chains, followed by formylation or acylation of the resulting hydroxylamines. While the oxidations are catalyzed by flavin-dependent monooxygenases [12–16], the carbon transfer is mediated either by tetrahydrofolate-dependent formyl [17] or by acyl-CoA-dependent acyl transferases [13,15–16]. It is widely assumed that the oxidation precedes the formylation or acylation [17–22]. In this study, however, two molecules were isolated (**1** and **3**), the structures of which suggest that the timing of the enzymatic reactions can be interchanged. An alternative explanation would be that compound **5** is metabolized to **1**, even though we did not find any literature evidence for such a reductive degradation of amamistatin-type siderophores.

Compound **6** represents a possible shunt product of amamistatin biosynthesis. A similar molecule and its putative biosynthetic pathway were recently described by Jaspars et al. [12]. In accordance with this proposal, salicylic acid and ʟ-threonine would form a salimethyloxazolinyl-thioester intermediate, which then undergoes a C–N bond opening resulting in an ester formation. Eventually, the thioester undergoes a hydrolysis reaction leading to compound **6**.

The compounds, which were discovered in this study, are members of the large family of nocobactin-type siderophores. Structurally, compounds **1**–**5** are closely related to formobactin [10], brasilibactin [23], nocardimicin [24], and terpenibactins [15]. While the core scaffold of these natural products is conserved, they differ in the length of their fatty acid residues, their methylation pattern, and the presence of an oxazole or an oxazoline ring. Up to now, it is not clear whether these structural variations are biologically relevant in terms of iron sequestration and cellular uptake.

## Conclusion

In summary, *Nocardia altamirensis* was found to synthesize various amamistatins under iron-deficient conditions. The structures of these natural products were verified by high-resolution mass spectrometry, tandem mass spectrometry as well as 1D and 2D NMR analyses. Their absolute configuration is consistent with the already known amamistatin B according to a comparison of optical rotation values and NOE data. The newly found compounds are assumed to represent intermediates or shunt products in amamistatin biosynthesis. In comparison to amamistatin B, they exhibit a lower iron affinity, which can be ascribed to the lack of hydroxamate groups.

## Experimental

### Analytical methods

LC–MS analysis was performed on a compact quadrupole-time of flight (Q-TOF) mass spectrometer from Bruker Daltonics with an Agilent 1260 Infinity LC system equipped with a Nucleoshell RP18 column (150 × 2.0 mm, Macherey-Nagel). NMR spectra were recorded on a Bruker 600 MHz Avance III HD system with methanol-*d*_4_ as solvent and internal standard. The solvent signal was referenced to δ_H_ 3.31 ppm and δ_C_ 49.0 ppm, respectively. Optical rotation was measured at 20 °C on a PerkinElmer polarimeter 341 with a sodium lamp (wavelength = 589 nm) using a 1 dm cuvette. For this, samples were dissolved in 1 mL methanol.

### Cultivation and extraction of *Nocardia altamirensis*

For siderophore production, the strain was grown in 5-L Erlenmeyer flasks containing 1 L of iron-free M9 medium: 7.52 g/L Na_2_HPO_4_×2H_2_O, 3.0 g/L KH_2_PO_4_, 0.5 g/L NaCl, 0.5 g/L NH_4_Cl, 4 g/L glucose, 0.247 g/L MgSO_4_×7H_2_O, 0.074 g CaCl_2_×2H_2_O, 1 mL biotin (1 mg/mL), 1 mL thiamin·HCl (1 mg/mL), 10 mL trace elements solution (0.05 g/L EDTA, 0.84 mg/L ZnCl_2_, 0.13 mg/L CuCl_2_×2H_2_O, 0.01 mg/L CoCl_2_×2H_2_O, 0.01 mg/L H_3_BO_3_, 0.0016 mg/L MnCl_2_×4H_2_O). The cultivation was conducted on a rotary shaker at 130 rpm and 30 °C for three weeks. Afterwards, adsorber resin (XAD-7, 20 g/L) was added to the culture broth to bind the secreted metabolites. The resin was separated from the culture broth by filtration, washed with distilled water, and exhaustively extracted with methanol.

### Isolation of metabolites **1**–**6**

The concentrated extract was first fractionated by flash column chromatography over Polygoprep 60-50 C_18_ (Macherey-Nagel) using an increasing concentration of methanol in water. Fractions that gave a color change in the CAS assay were prepurified by reversed-phase HPLC using a Nucleodur C18 ec column (125 × 21 mm, 5 μm, Macherey-Nagel) and a gradient of methanol in water supplemented with 0.1% (v/v) trifluoroacetic acid. The gradient conditions were as follows: from 40% methanol to 70% in 5 minutes followed by an increase to 90% in 20 minutes, kept at 90% for 10 minutes. The flow rate was set to 10 mL/min. The elution of compounds was monitored with a diode array detector. Again, CAS active fractions were collected. For the final purification step, a Nucleodur C18 Isis column (250 × 10 mm, 5 μm, Macherey-Nagel) was used with a linear gradient from 25% to 90% acetonitrile in water + 0.1% (v/v) trifluoroacetic acid over a period of 30 min. The flow rate was set to 6 mL/min. The elution of compounds was monitored with a diode array detector.

### Chrome azurol S (CAS) assay

The CAS assay solution was prepared as previously described [9]. This solution was aliquoted (100 μL) into the wells of a 96-well flat-bottomed microtiter plate. Subsequently, 5 μL test solution (= serial dilution of compounds **1**–**6** dissolved in DMSO) were added to every well. EDTA was used as a reference and dissolved in water. The microtiter plate was gently shaken for 4 h at room temperature. After this incubation period the remaining CAS–Fe^3+^ complex in each well was quantified by measuring the absorbance at 630 nm in a microplate reader. The values were plotted and the DC_50_ values were calculated. All tests were run in triplicate.

## Supporting Information

File 1 Copies of MS/MS and NMR spectra for compounds **1**–**6**.
