# Supplementary material for: Amamistatins isolated from Nocardia altamirensis
Source: Beilstein J Org Chem. 2022 Mar 30;18:360–7. doi: 10.3762/bjoc.18.40 (PMC8978914; doi:10.3762/bjoc.18.40)
Supplement: File 1 — Copies of MS/MS and NMR spectra for compounds 1–6. [file Beilstein_J_Org_Chem-18-360-s001.pdf]

BEILSTEIN JOURNAL OF ORGANIC CHEMISTRY

Supporting Information

for

Amamistatins isolated from *Nocardia altamirensis*

Till Steinmetz, Wolf Hiller and Markus Nett

Beilstein J. Org. Chem. **2022,** *18,* 360–367. doi:10.3762/bjoc.18.40

Copies of MS/MS and NMR spectra for compounds 1–6

License and Terms: This is a supporting information file under the terms of the Creative Commons Attribution License (https://creativecommons.org/licenses/by/4.0). Please note that the reuse, redistribution and reproduction in particular requires that the author(s) and source are credited and that individual graphics may be subject to special legal provisions.
The license is subject to the *Beilstein Journal of Organic Chemistry* terms and conditions: (https://www.beilstein-journals.org/bjoc/terms)

Table of contents

Figure S1: MS/MS spectrum of **1** S2
Figure S2: ^{1}H NMR spectrum of **1** (600 MHz, methanol-d_4, 25 °C) S3
Figure S3: ^{13}C NMR spectrum of **1** (150 MHz, methanol-d_4, 25 °C) S4
Figure S4: COSY spectrum of **1** (600 MHz, methanol-d_4, 25 °C) S5
Figure S5: HSQC spectrum of **1** (600 MHz, methanol-d_4, 25 °C) S6
Figure S6: HMBC spectrum of **1** (600 MHz, methanol-d_4, 25 °C) S7
Figure S7: MS/MS spectrum of **2** S8
Figure S8: ^{1}H NMR spectrum of **2** (600 MHz, methanol-d_4, 25 °C) S9
Figure S9: ^{13}C NMR spectrum of **2** (150 MHz, methanol-d_4, 25 °C) S10
Figure S10: COSY spectrum of **2** (600 MHz, methanol-d_4, 25 °C) S11
Figure S11: HSQC spectrum of **2** (600 MHz, methanol-d_4, 25 °C) S12
Figure S12: HMBC spectrum of **2** (600 MHz, methanol-d_4, 25 °C) S13
Figure S13: MS/MS spectrum of **3** S14
Figure S14: ^{1}H NMR spectrum of **3** (600 MHz, methanol-d_4, 25 °C) S15
Figure S15: ^{13}C NMR spectrum of **3** (150 MHz, methanol-d_4, 25 °C) S16
Figure S16: COSY spectrum of **3** (600 MHz, methanol-d_4, 25 °C) S17
Figure S17: HSQC spectrum of **3** (600 MHz, methanol-d_4, 25 °C) S18
Figure S18: HMBC spectrum of **3** (600 MHz, methanol-d_4, 25 °C) S19
Figure S19: MS/MS spectrum of **4** S20
Figure S20: ^{1}H NMR spectrum of **4** (600 MHz, methanol-d_4, 25 °C) S21
Figure S21: ^{13}C NMR spectrum of **4** (150 MHz, methanol-d_4, 25 °C) S22
Figure S22: COSY spectrum of **4** (600 MHz, methanol-d_4, 25 °C) S23
Figure S23: HSQC spectrum of **4** (600 MHz, methanol-d_4, 25 °C) S24
Figure S24: HMBC spectrum of **4** (600 MHz, methanol-d_4, 25 °C) S25
Figure S25: MS/MS spectrum of **5** S26
Figure S26: ^{1}H NMR spectrum of **5** (600 MHz, methanol-d_4, 25 °C) S27
Figure S27: ^{13}C NMR spectrum of **5** (150 MHz, methanol-d_4, 25 °C) S28
Figure S28: COSY spectrum of **5** (600 MHz, methanol-d_4, 25 °C) S29
Figure S29: HSQC spectrum of **5** (600 MHz, methanol-d_4, 25 °C) S30
Figure S30: HMBC spectrum of **5** (600 MHz, methanol-d_4, 25 °C) S31
Figure S31: MS/MS spectrum of **6** S32
Figure S32: ^{1}H NMR spectrum of **6** (600 MHz, methanol-d_4, 25 °C) S33
Figure S33: ^{13}C NMR spectrum of **6** (150 MHz, methanol-d_4, 25 °C) S34
Figure S34: COSY spectrum of **6** (600 MHz, methanol-d_4, 25 °C) S35
Figure S35: HSQC spectrum of **6** (600 MHz, methanol-d_4, 25 °C) S36
Figure S36: HMBC spectrum of **6** (600 MHz, methanol-d_4, 25 °C) S37

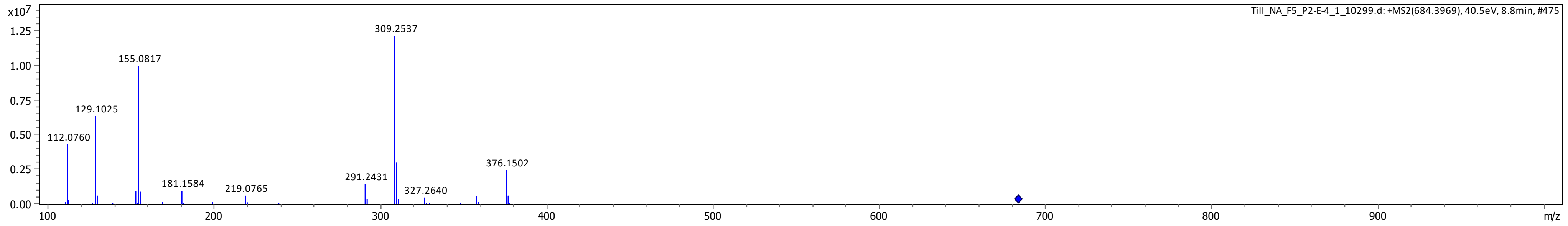

Figure S1: MS/MS spectrum of **1**

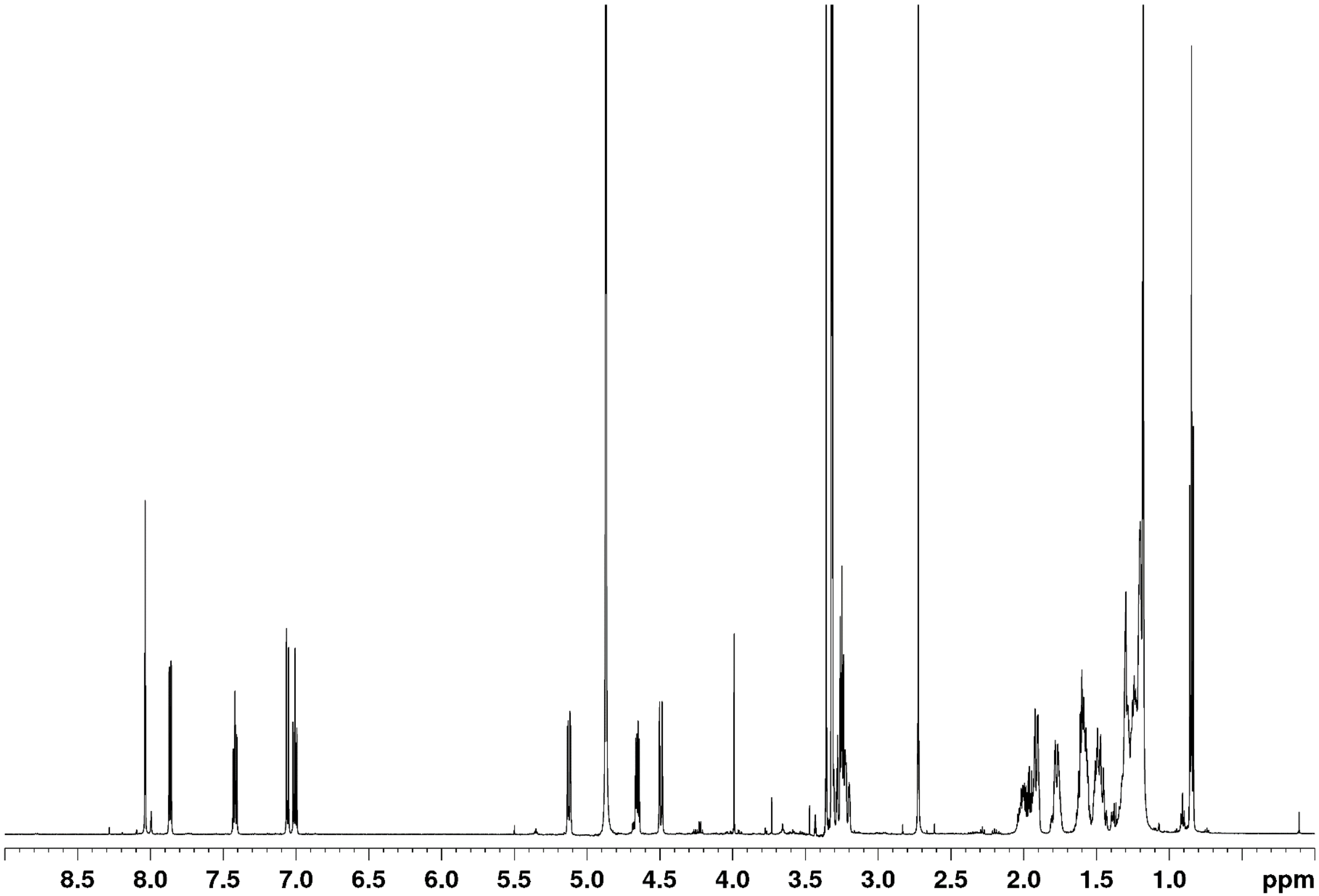

Figure S2: ^{1}H NMR spectrum of **1** (600 MHz, methanol-d_4, 25 °C)

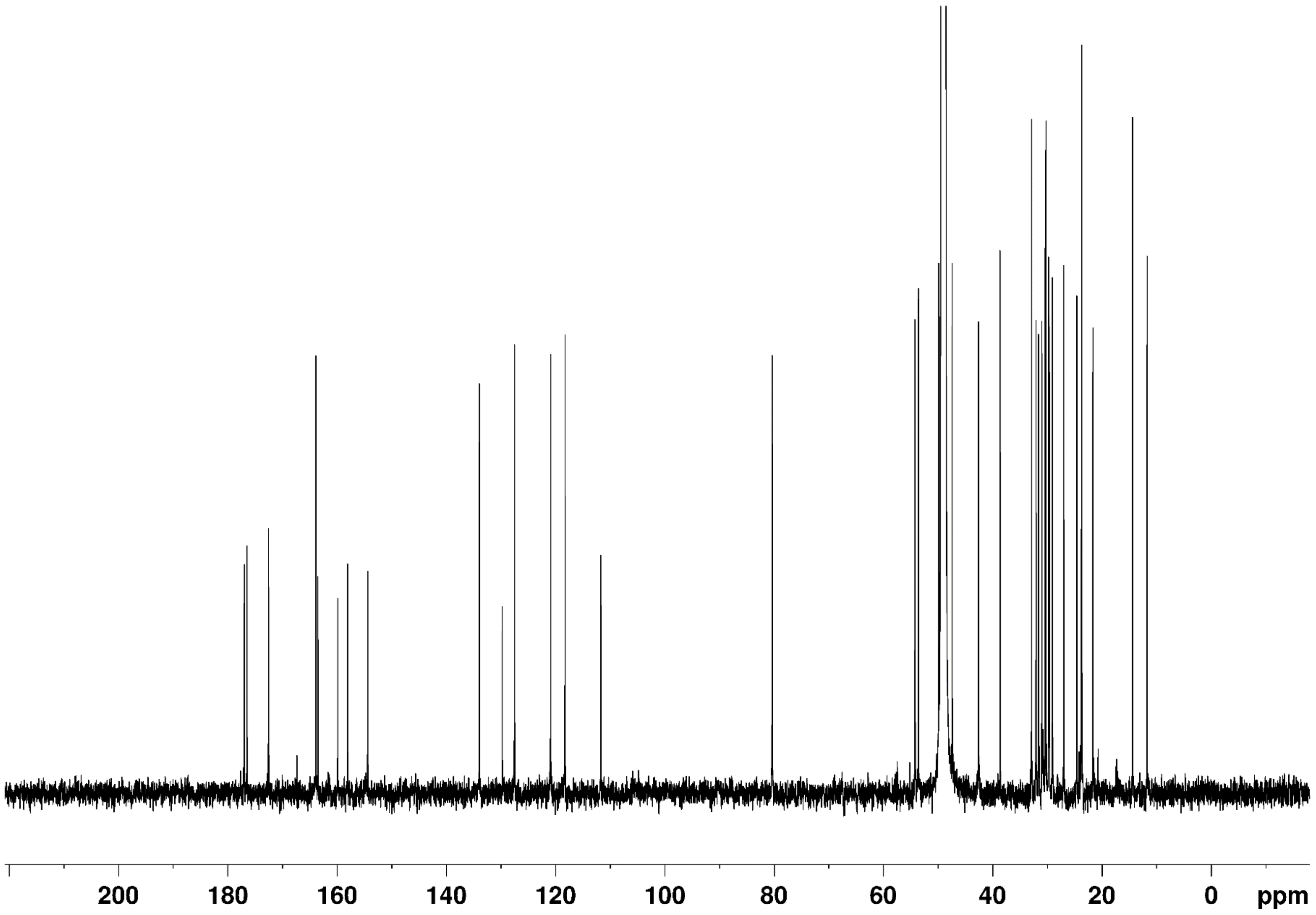

Figure S3: ^{13}C NMR spectrum of **1** (150 MHz, methanol-d_4, 25 °C)

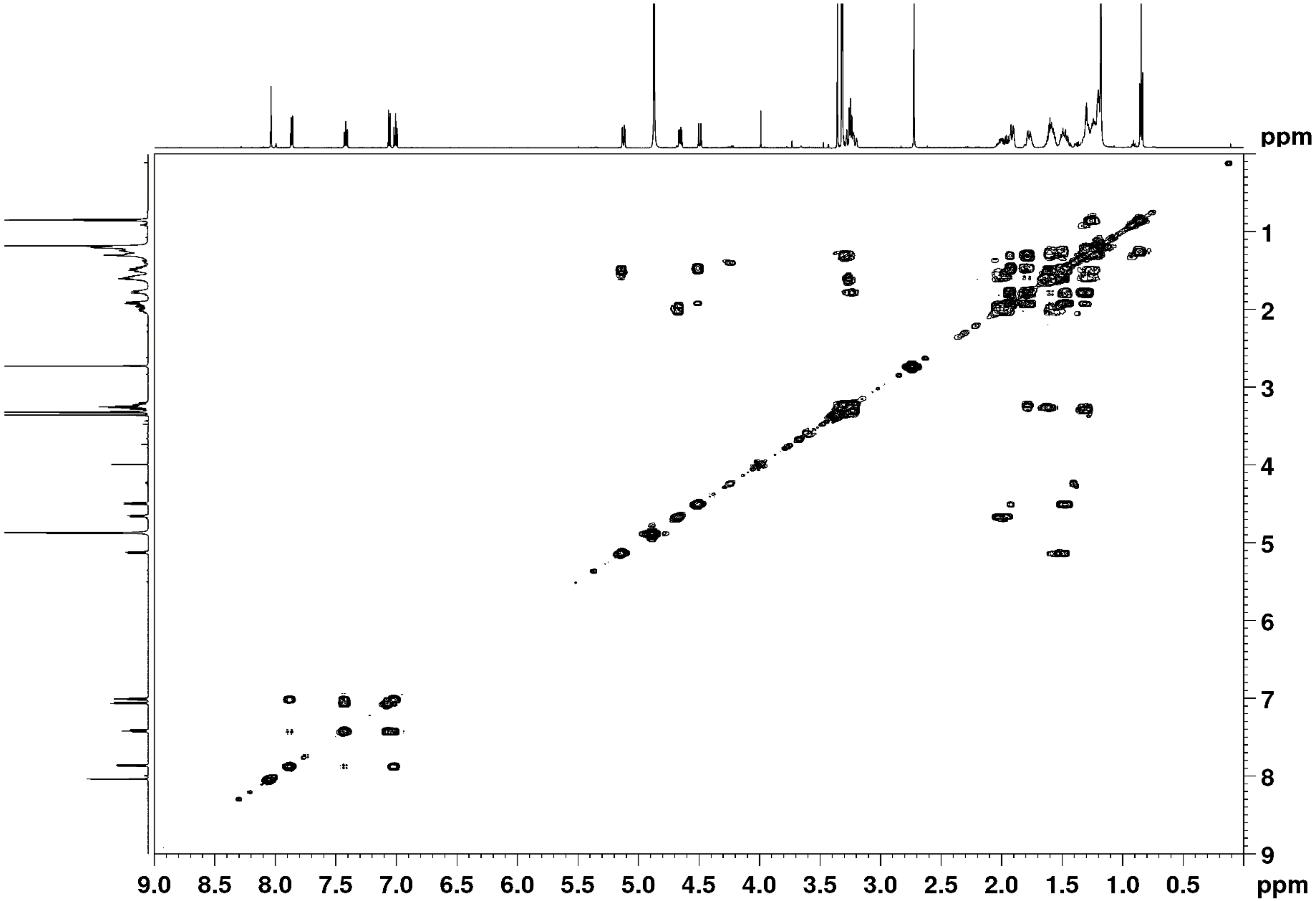

Figure S4: COSY spectrum of **1** (600 MHz, methanol-d_4, 25 °C)

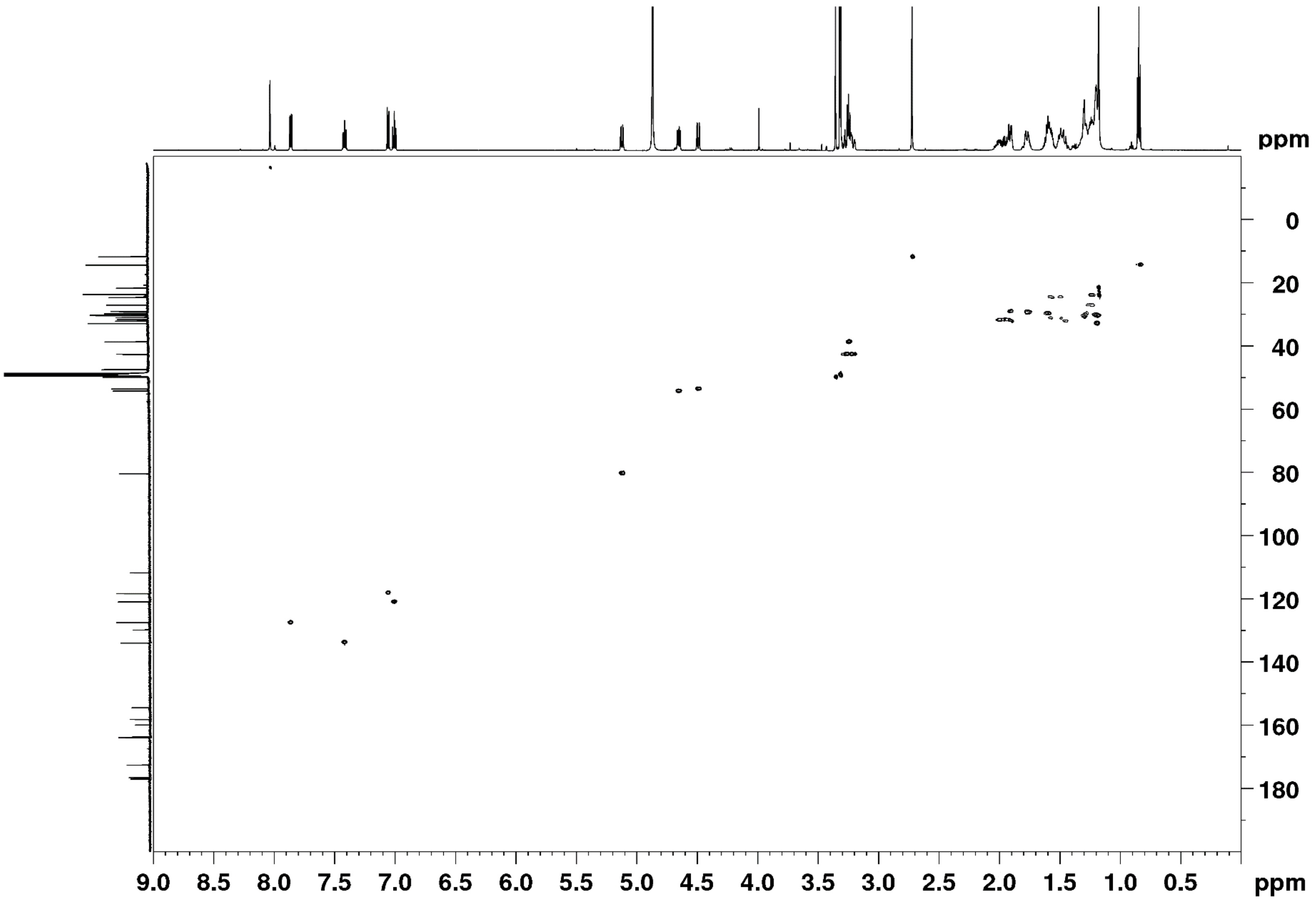

Figure S5: HSQC spectrum of **1** (600 MHz, methanol-d_4, 25 °C)

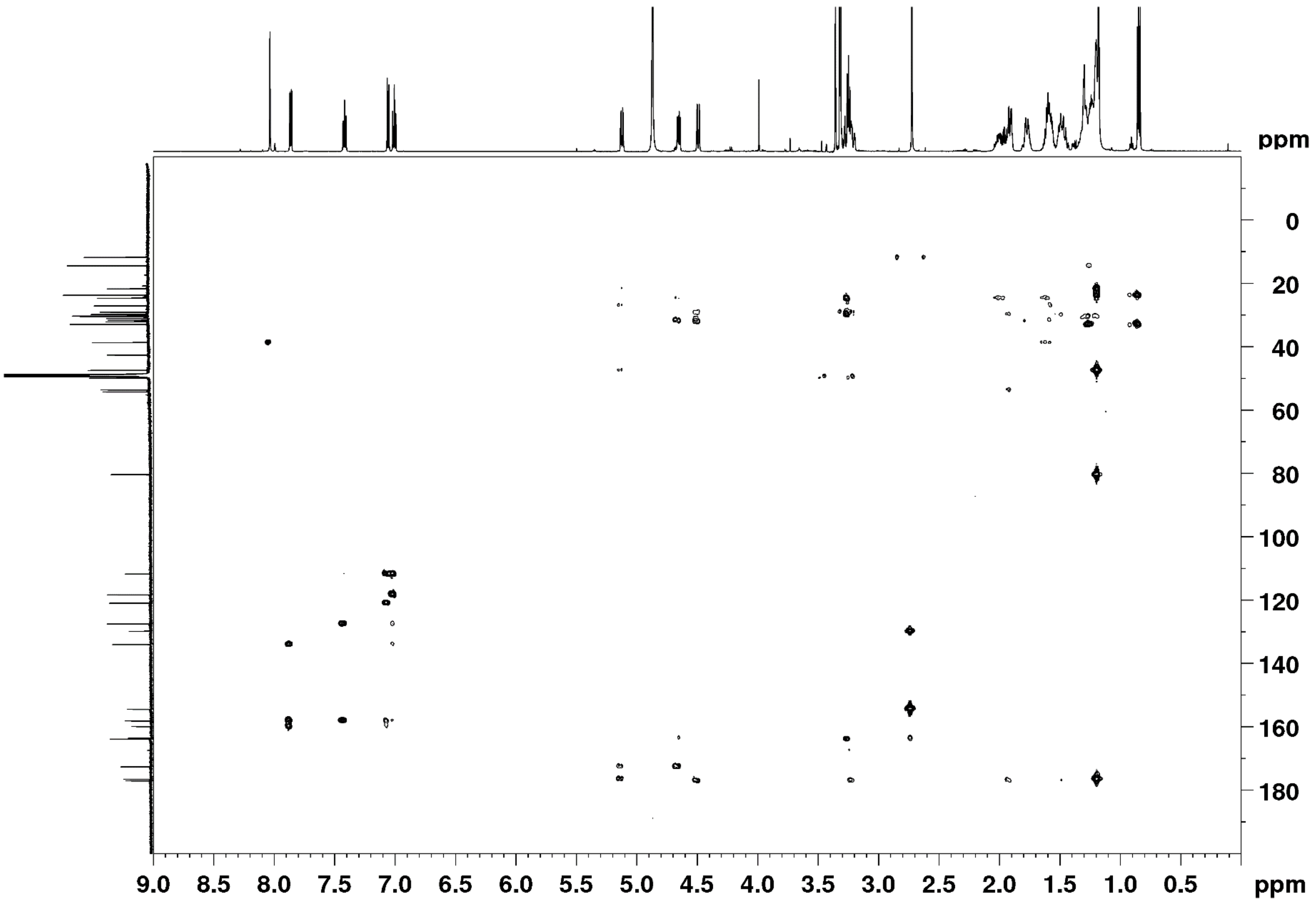

Figure S6: HMBC spectrum of **1** (600 MHz, methanol-d_4, 25 °C)

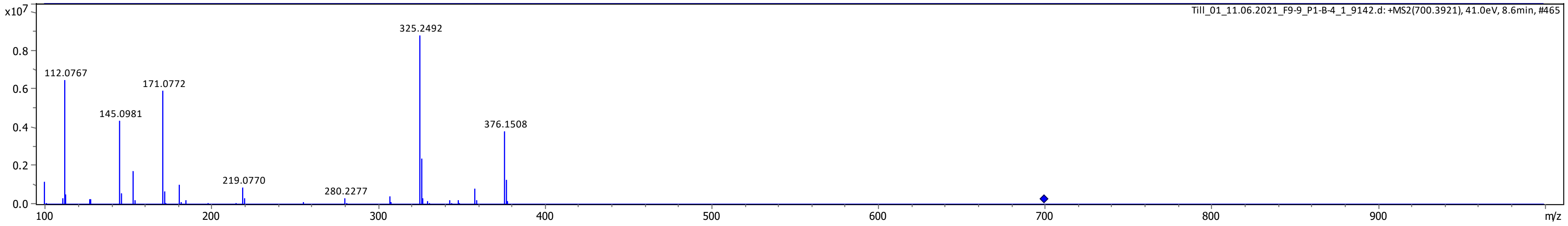

Figure S7: MS/MS spectrum of **2**

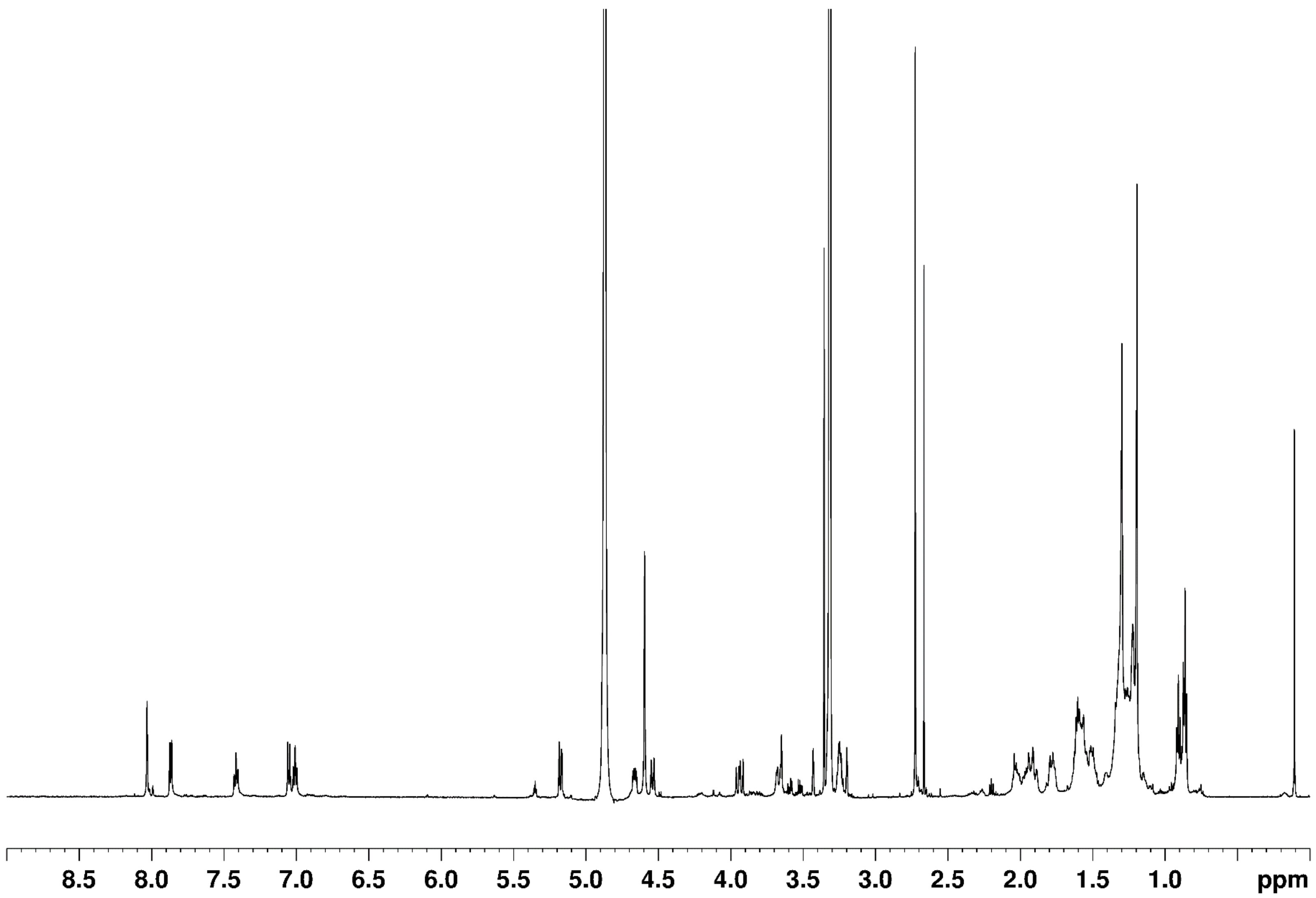

Figure S8: ^{1}H NMR spectrum of **2** (600 MHz, methanol-d_4, 25 °C)

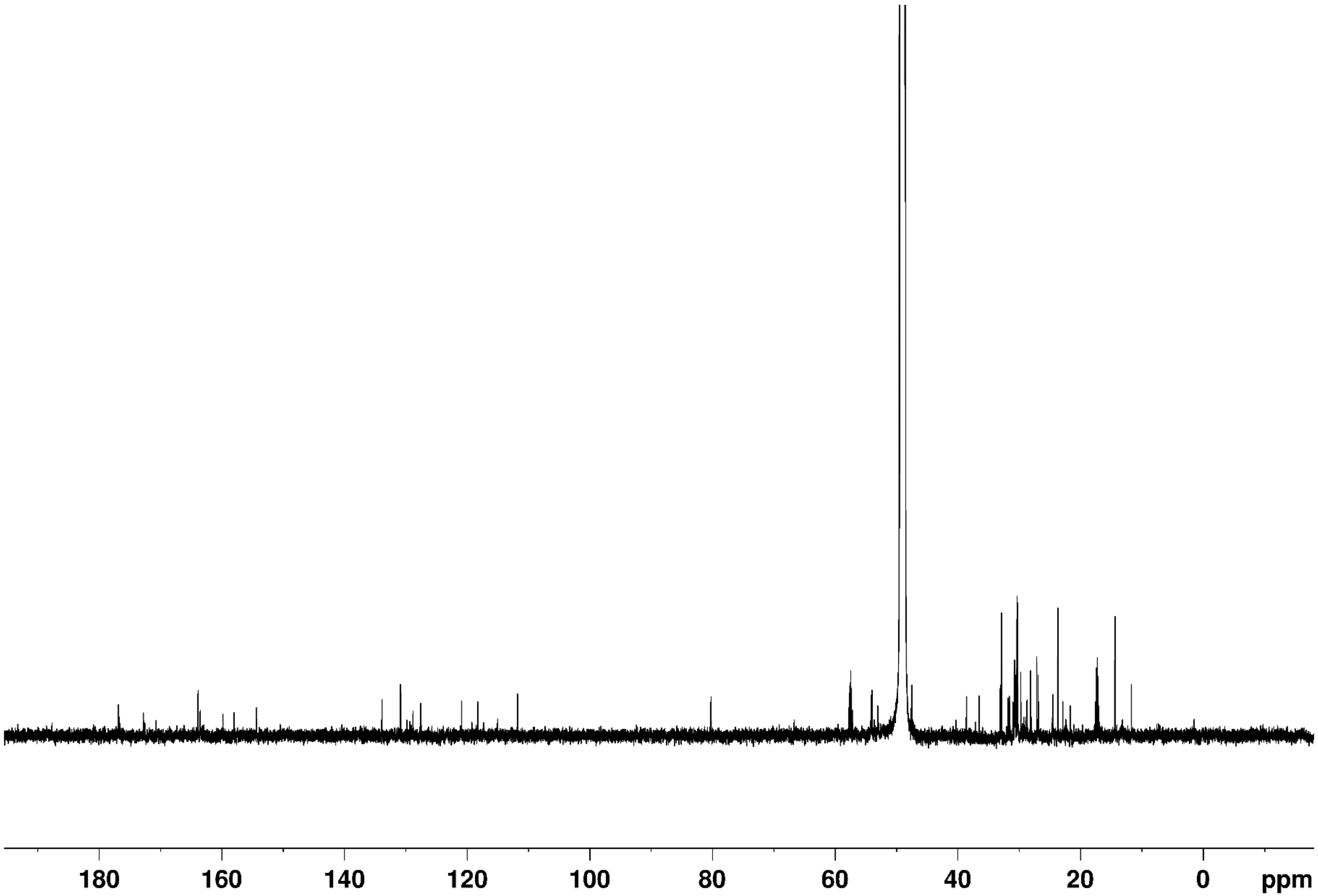

Figure S9: ^{13}C NMR spectrum of **2** (150 MHz, methanol-d_4, 25 °C)

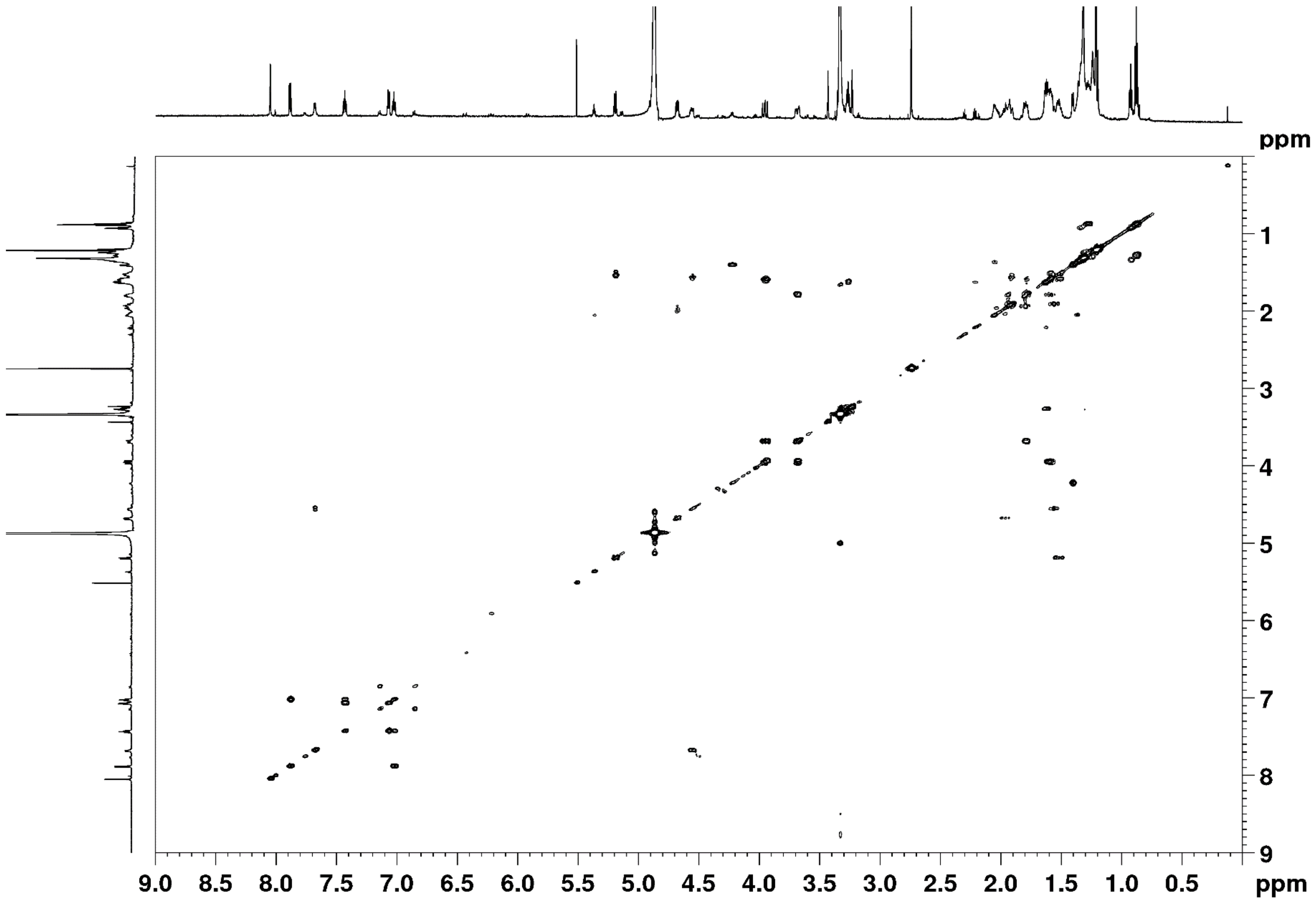

Figure S10: COSY spectrum of **2** (600 MHz, methanol-d_4, 25 °C)

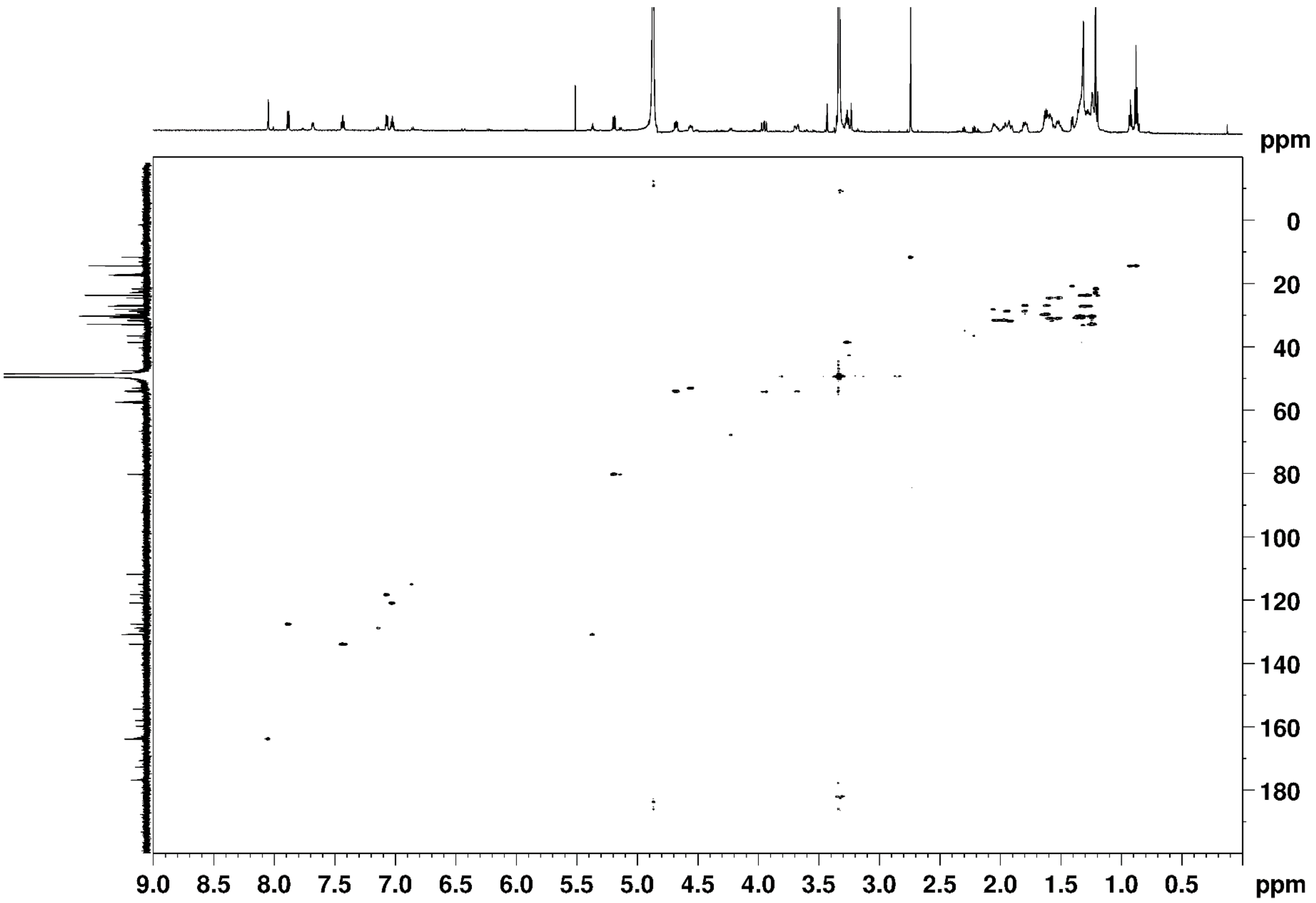

Figure S11: HSQC spectrum of **2** (600 MHz, methanol-d_4, 25 °C)

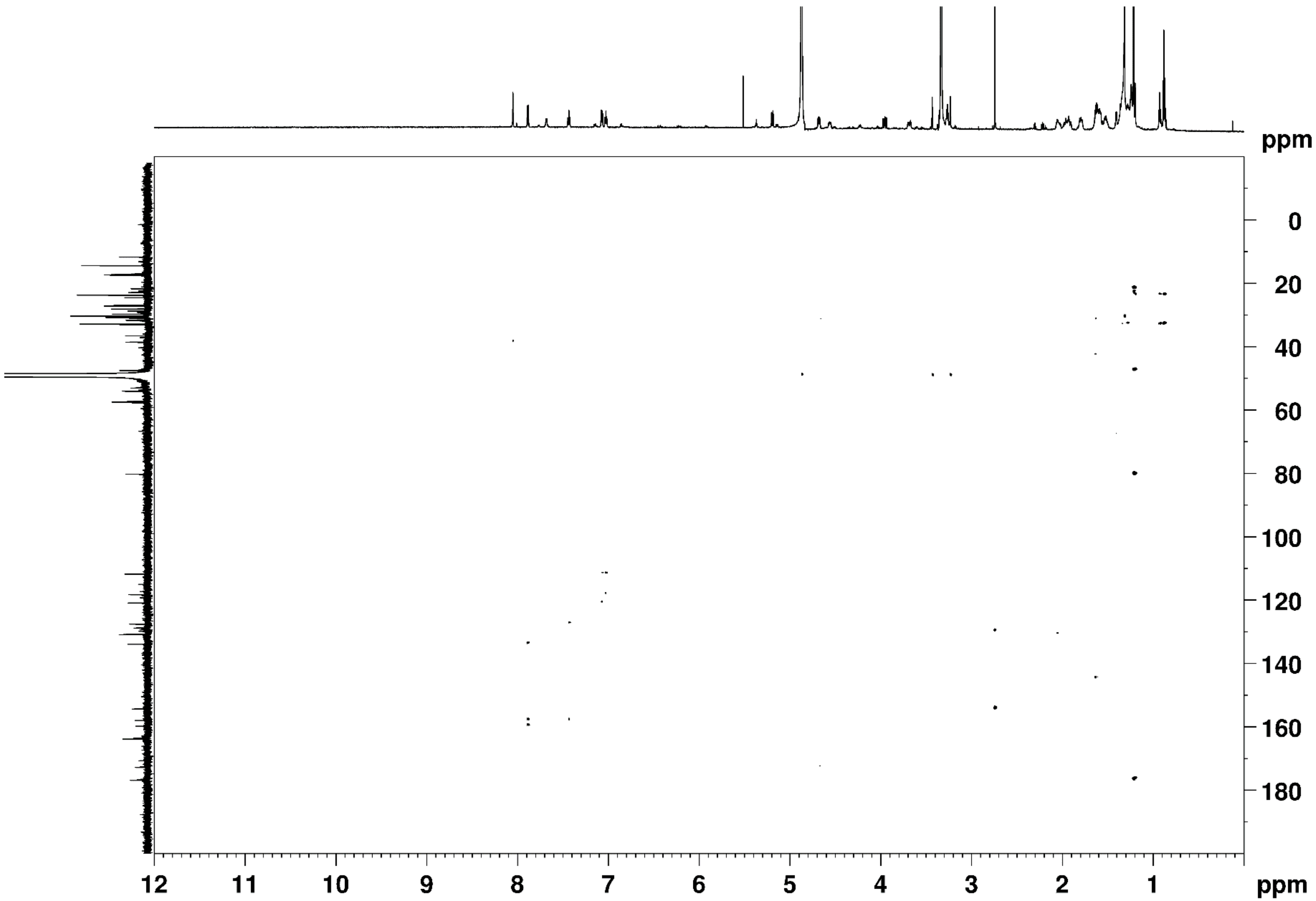

Figure S12: HMBC spectrum of **2** (600 MHz, methanol-d_4, 25 °C)

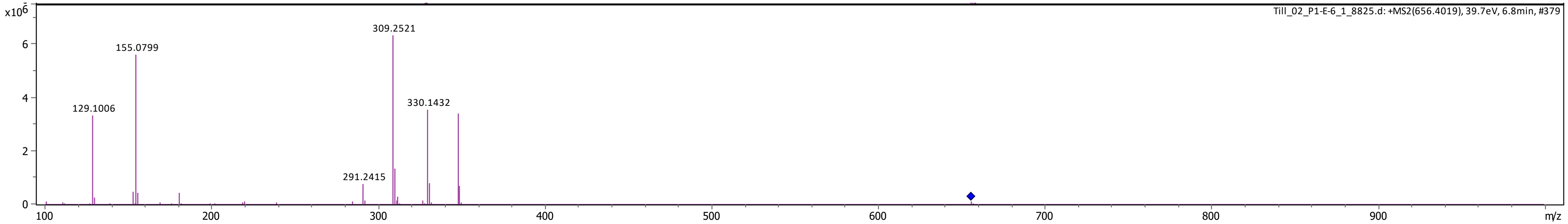

Figure S13: MS/MS spectrum of **3**

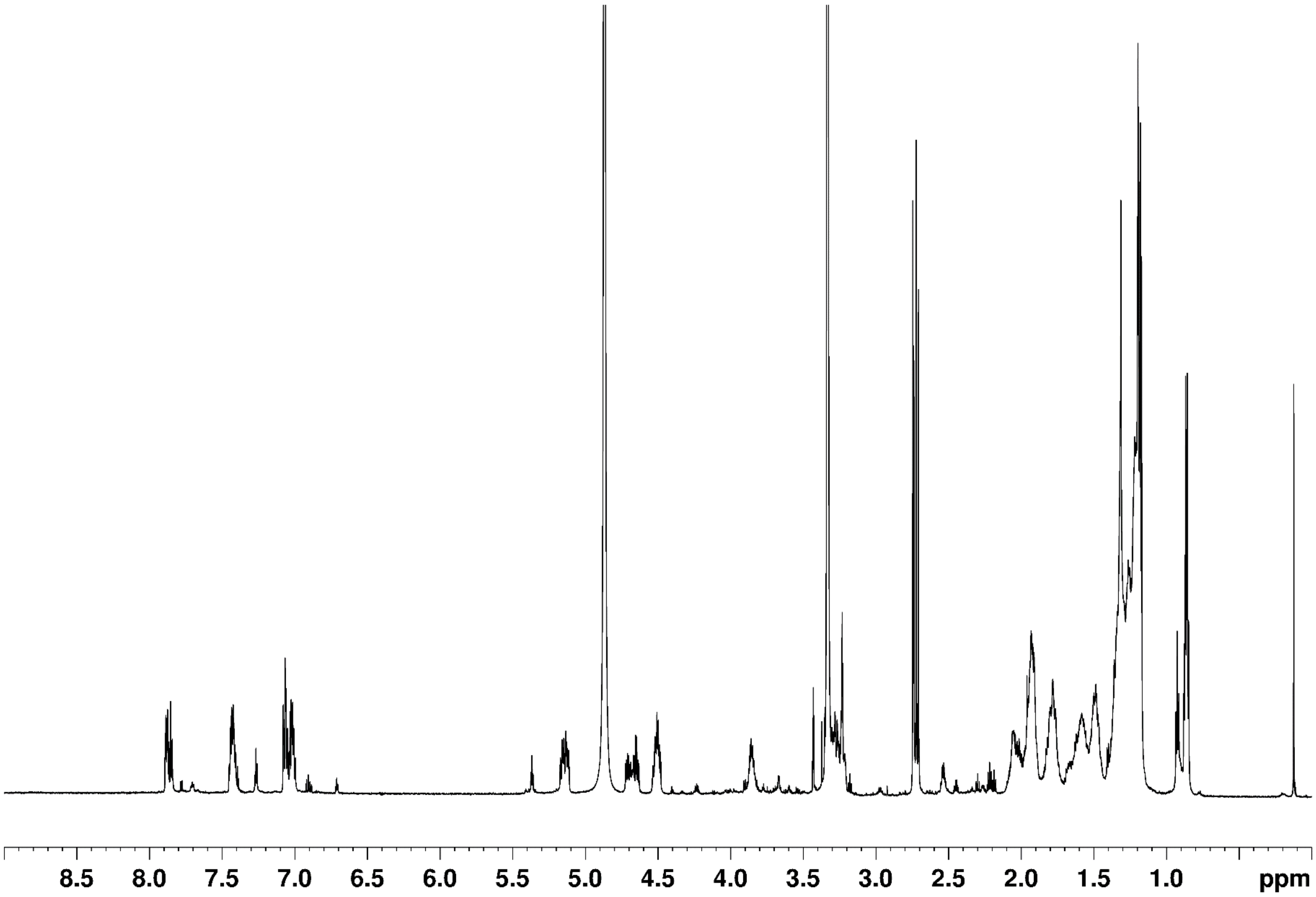

Figure S14: ^{1}H NMR spectrum of **3** (600 MHz, methanol-*d*$_4$, 25 °C)

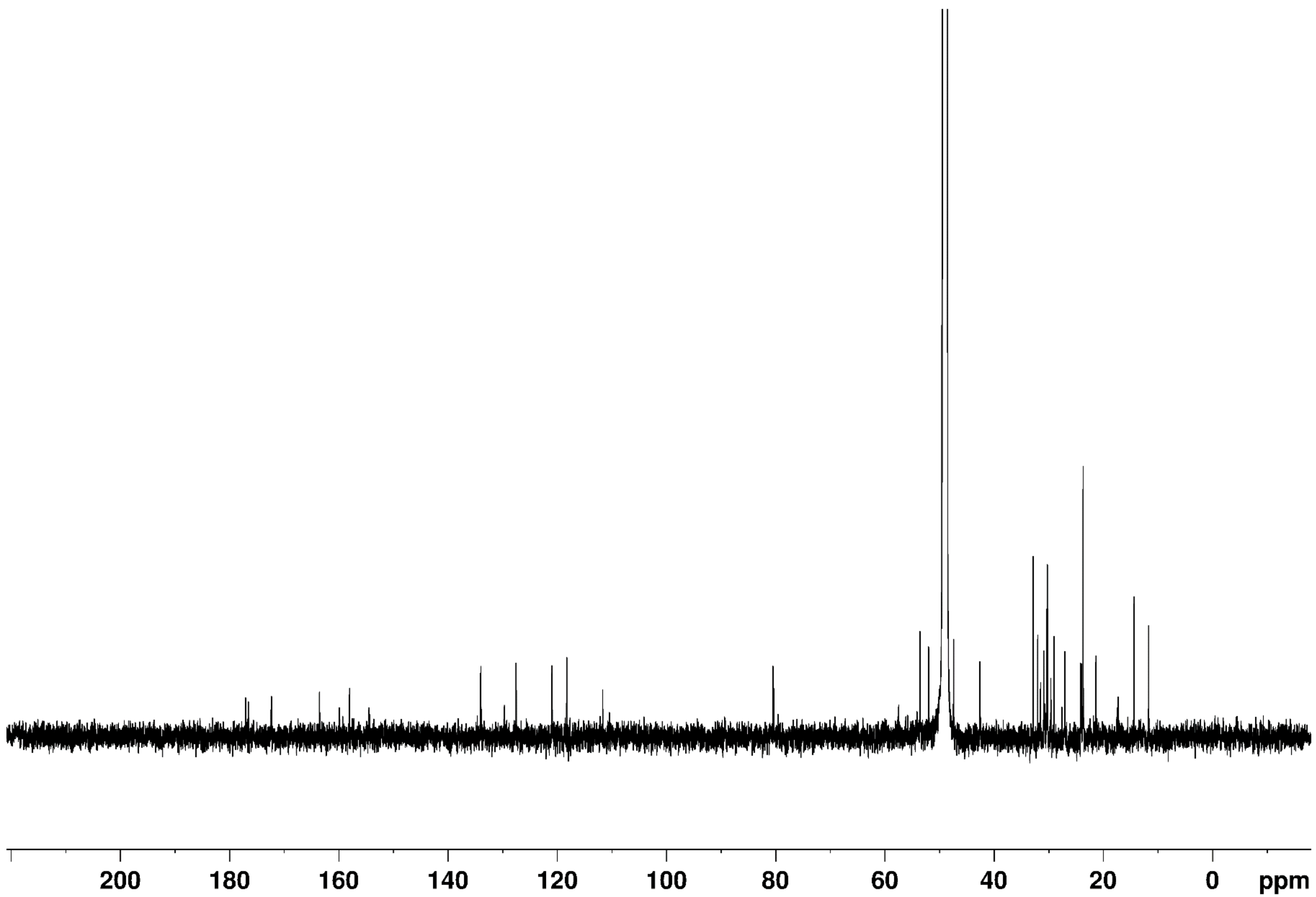

Figure S15: ^{13}C NMR spectrum of **3** (150 MHz, methanol-d_4, 25 °C)

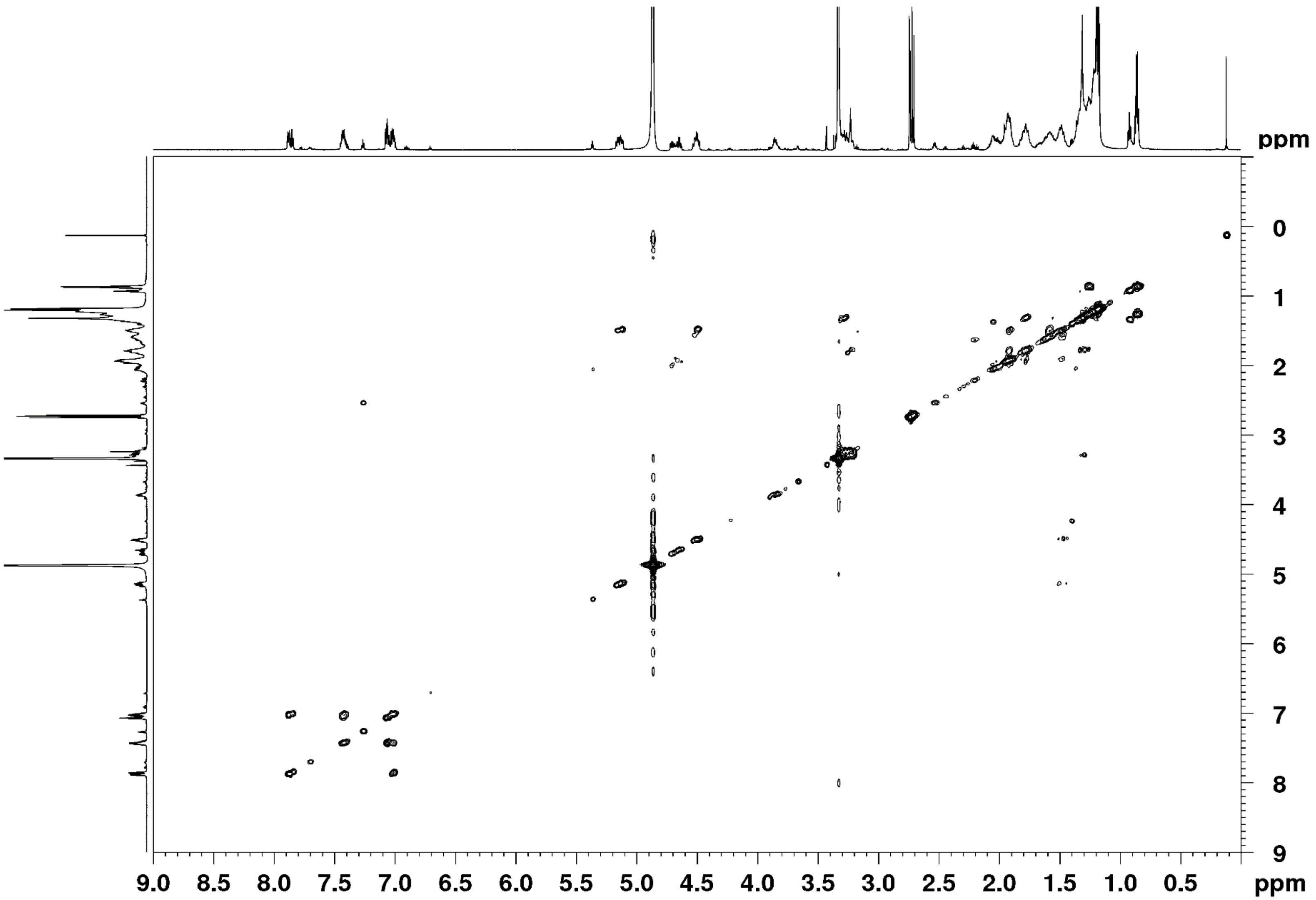

Figure S16: COSY spectrum of **3** (600 MHz, methanol-d_4, 25 °C)

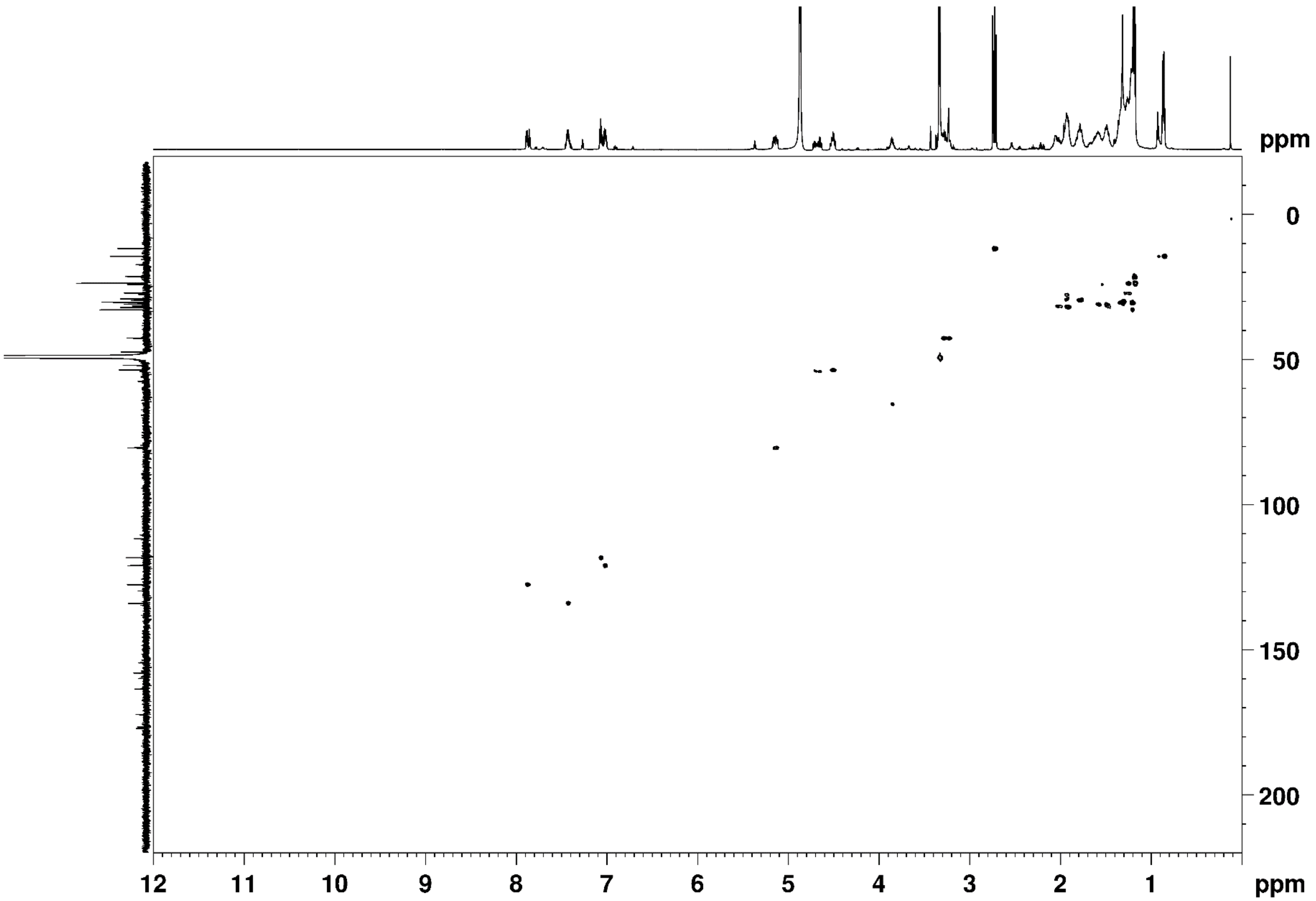

Figure S17: HSQC spectrum of **3** (600 MHz, methanol-d_4, 25 °C)

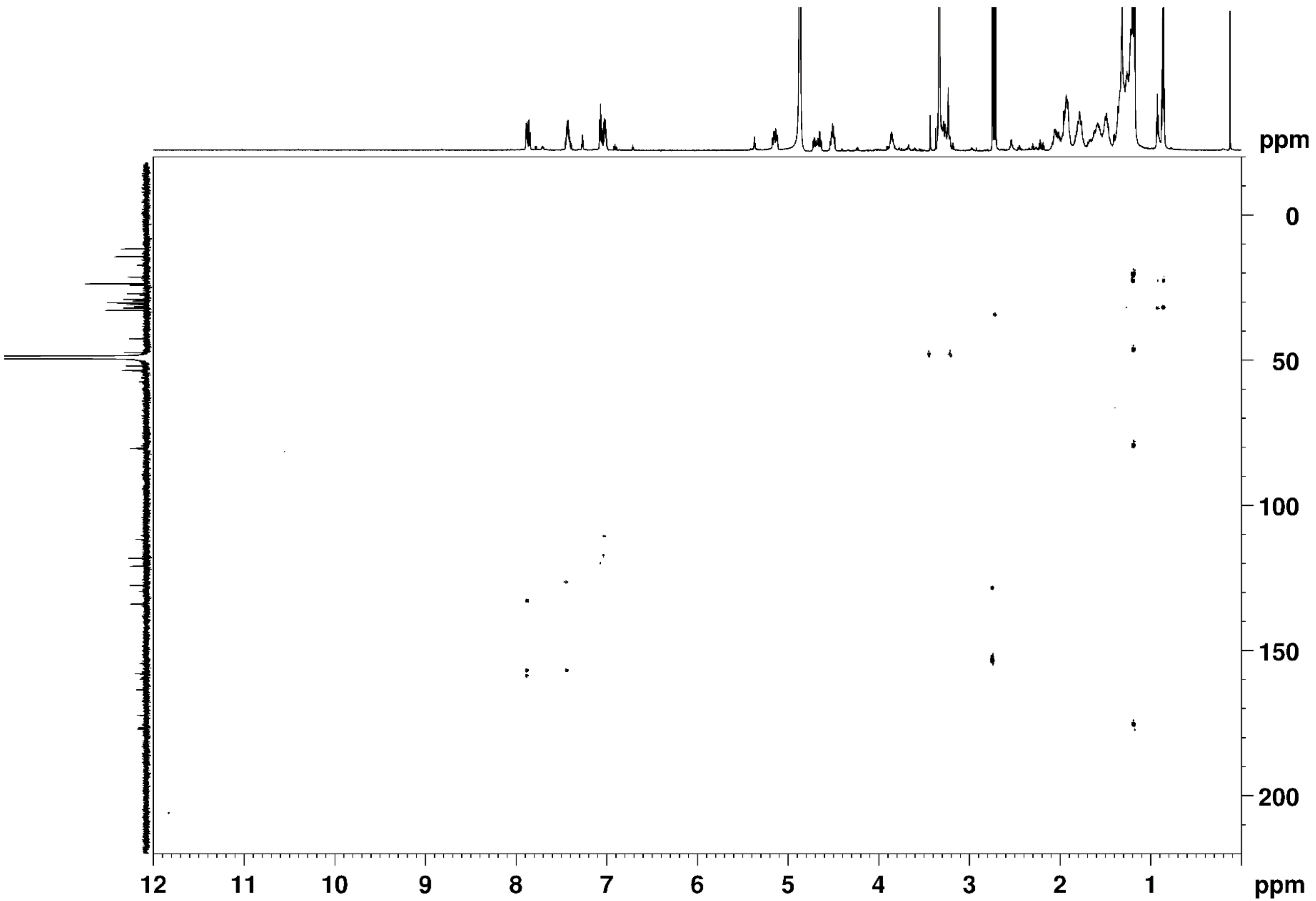

Figure S18: HMBC spectrum of **3** (600 MHz, methanol-d_4, 25 °C)

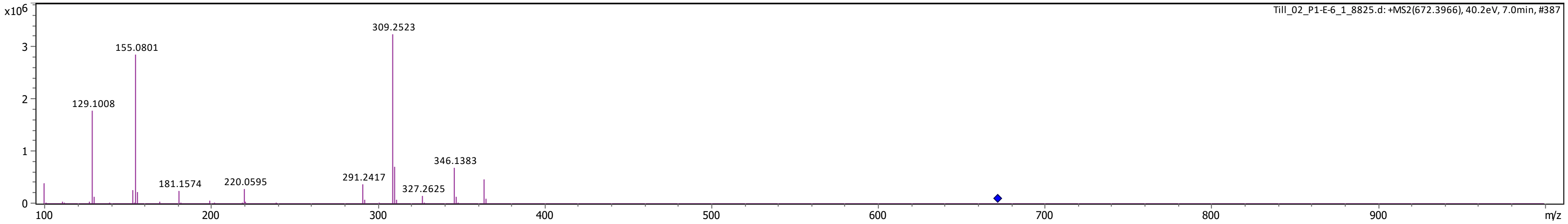

Figure S19: MS/MS spectrum of **4**

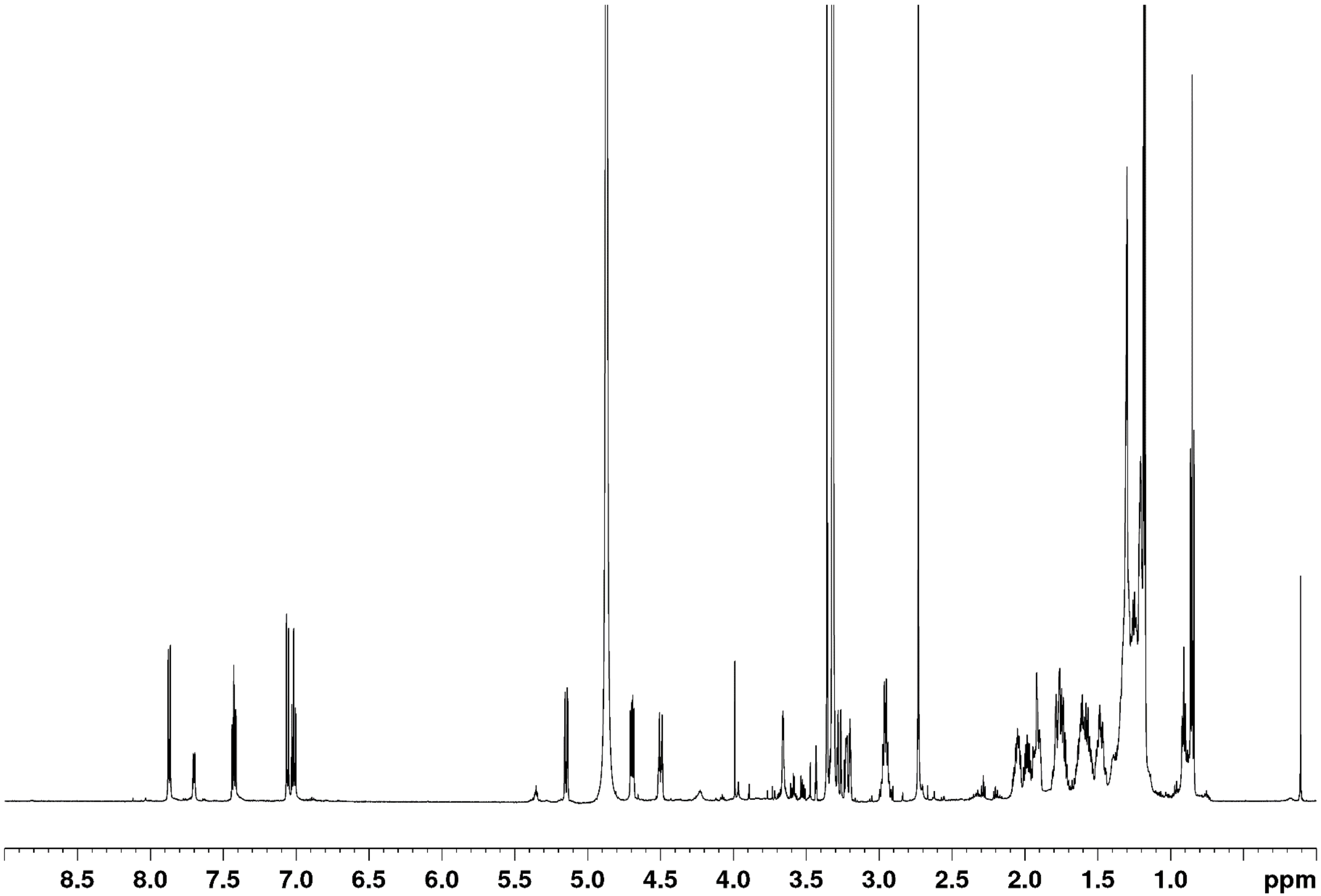

Figure S20: ^{1}H NMR spectrum of **4** (600 MHz, methanol-d_4, 25 °C)

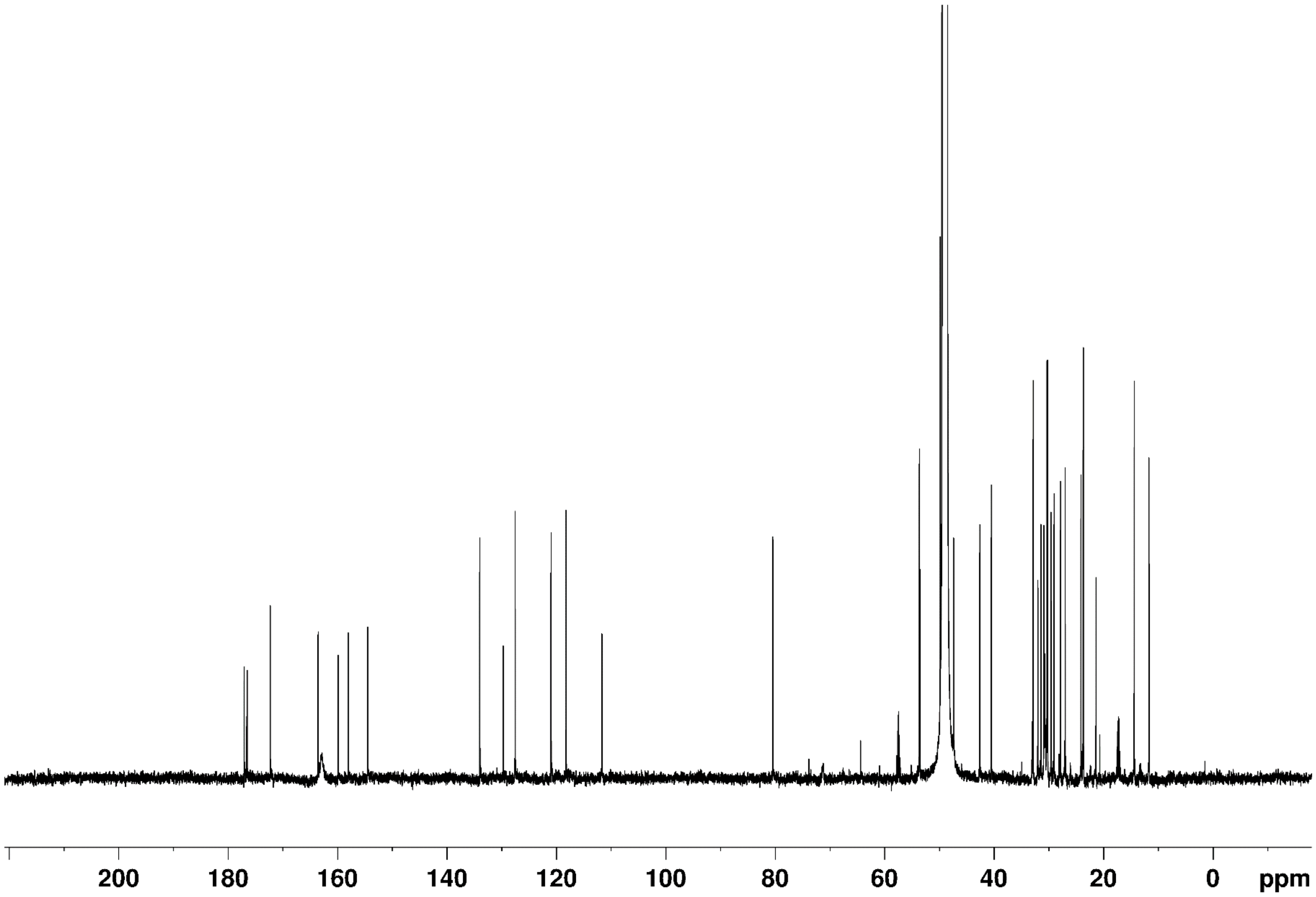

Figure S21: ^{13}C NMR spectrum of **4** (150 MHz, methanol-d_4, 25 °C)

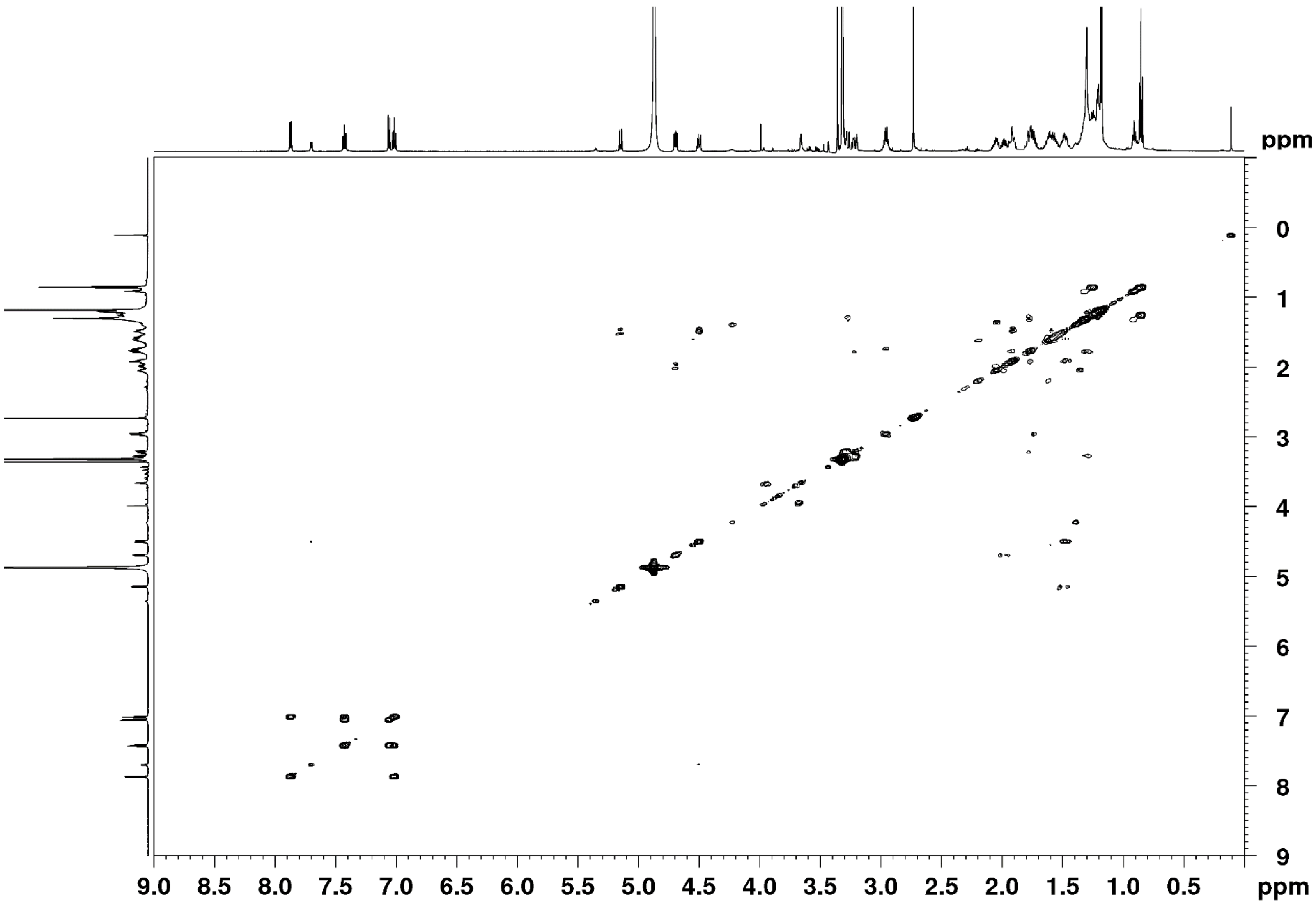

Figure S22: COSY spectrum of **4** (600 MHz, methanol-d_4, 25 °C)

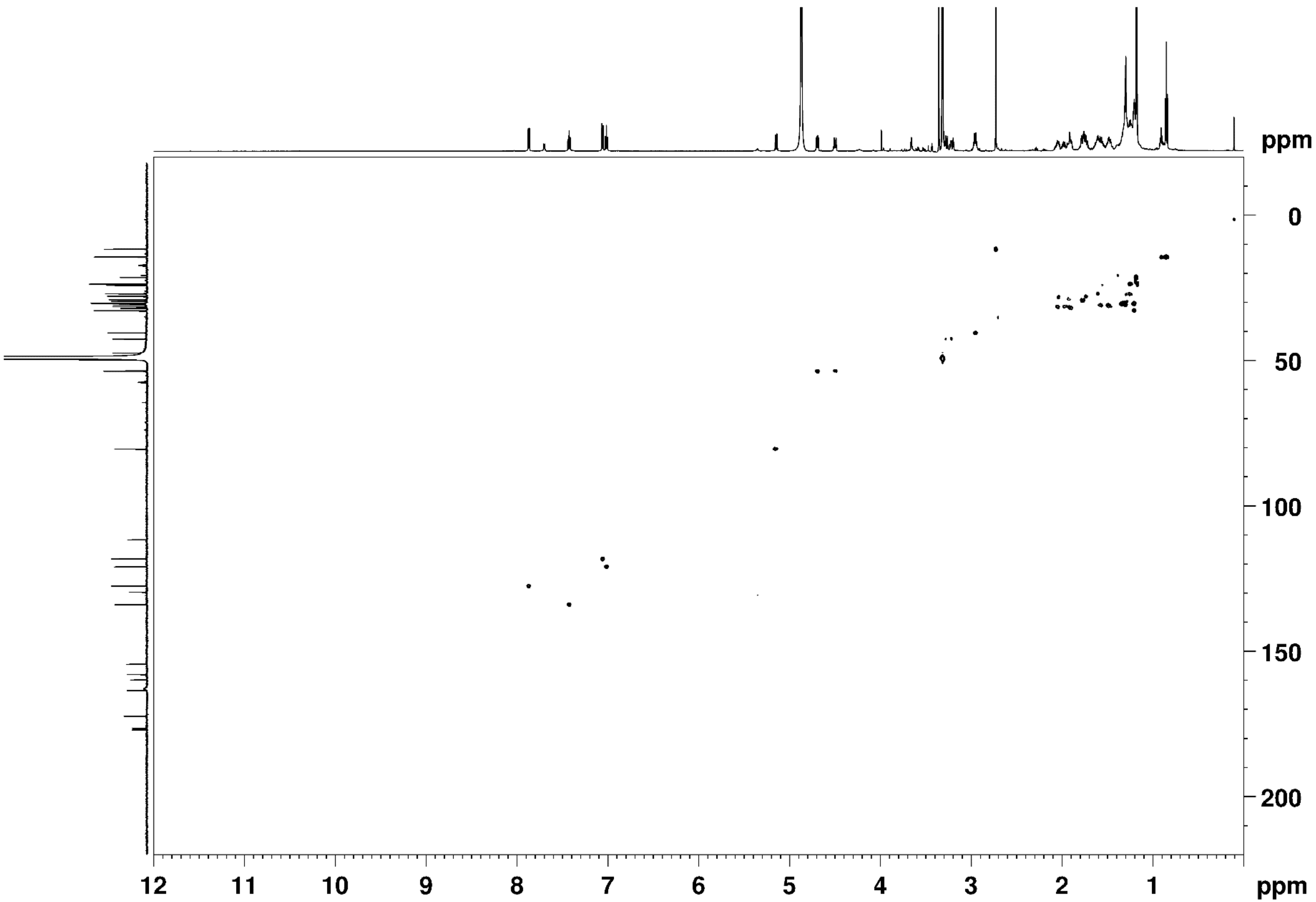

Figure S23: HSQC spectrum of **4** (600 MHz, methanol-d_4, 25 °C)

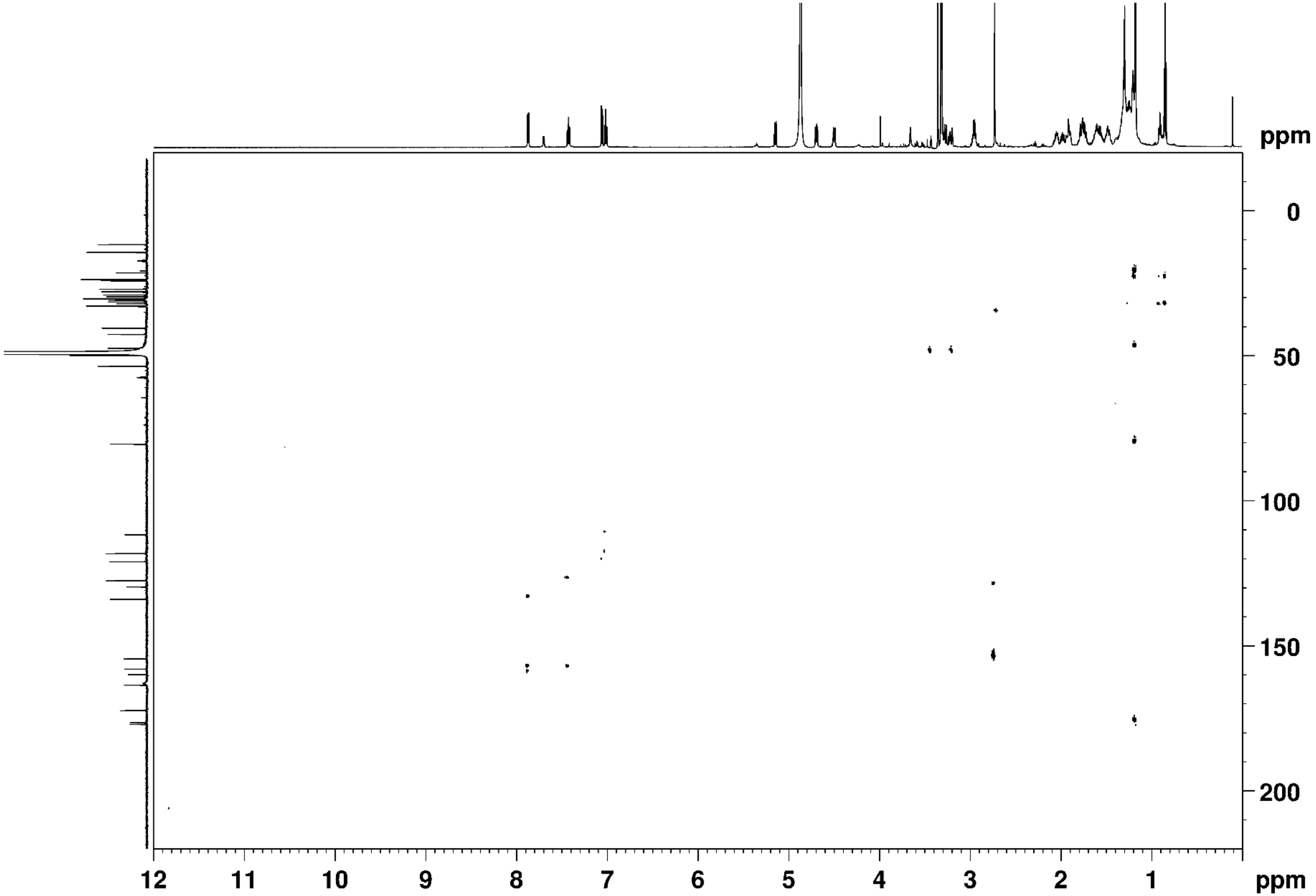

Figure S24: HMBC spectrum of **4** (600 MHz, methanol-d_4, 25 °C)

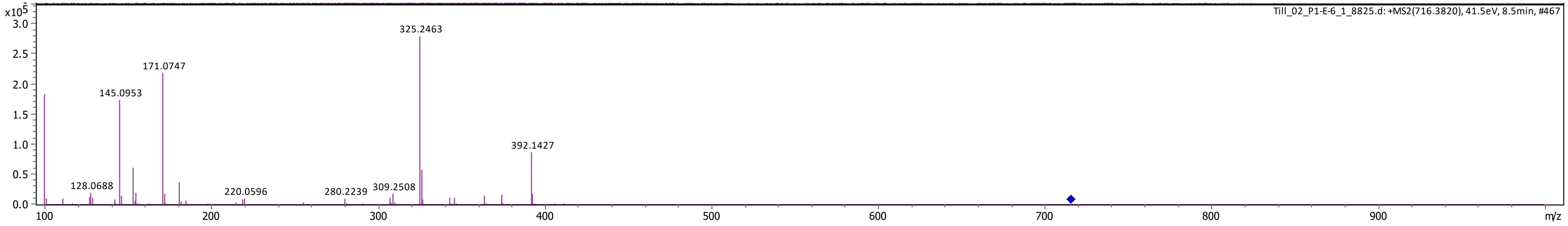

Figure S25: MS/MS spectrum of **5**

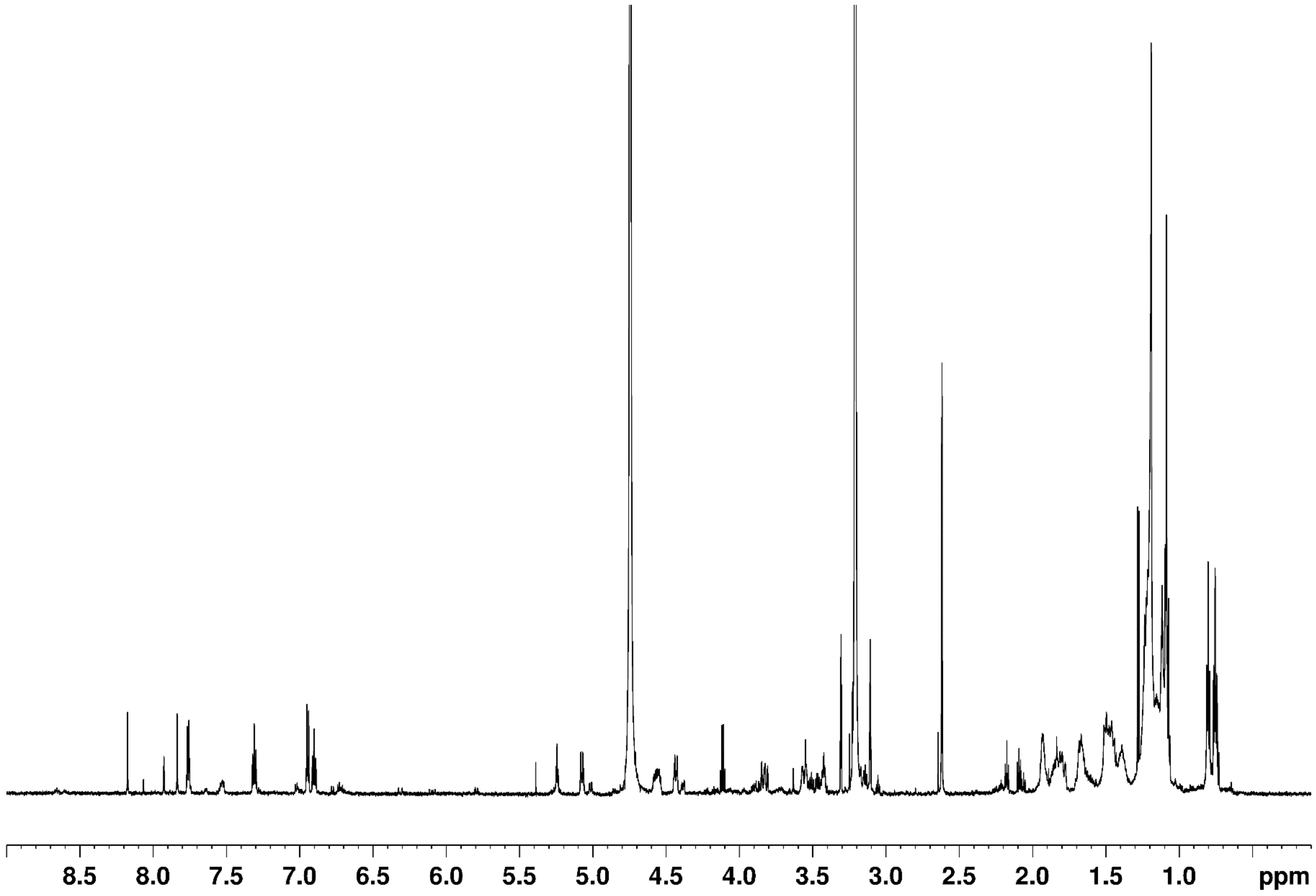

Figure S26: ^{1}H NMR spectrum of **5** (600 MHz, methanol-d_4, 25 °C)

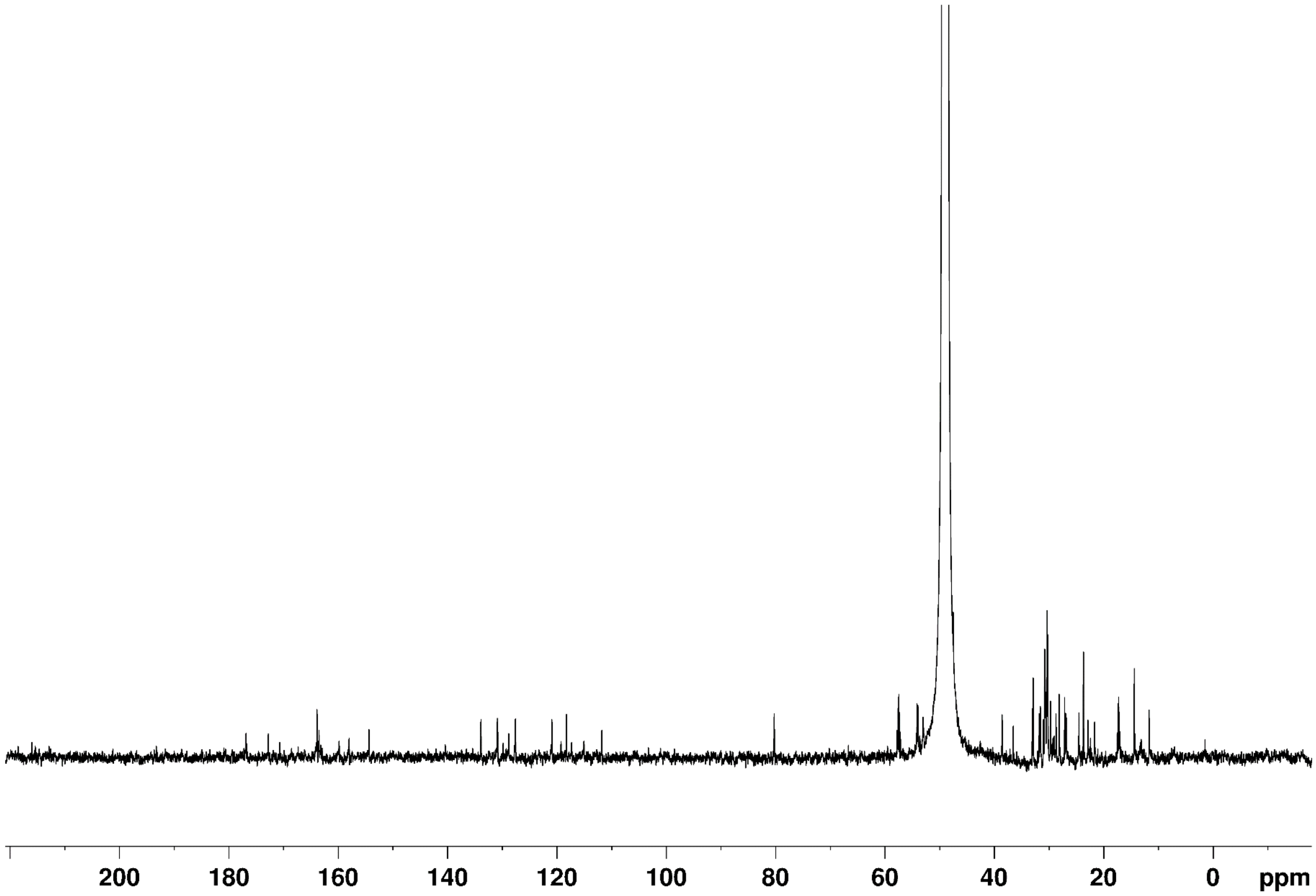

Figure S27: ^{13}C NMR spectrum of **5** (150 MHz, methanol-d_4, 25 °C)

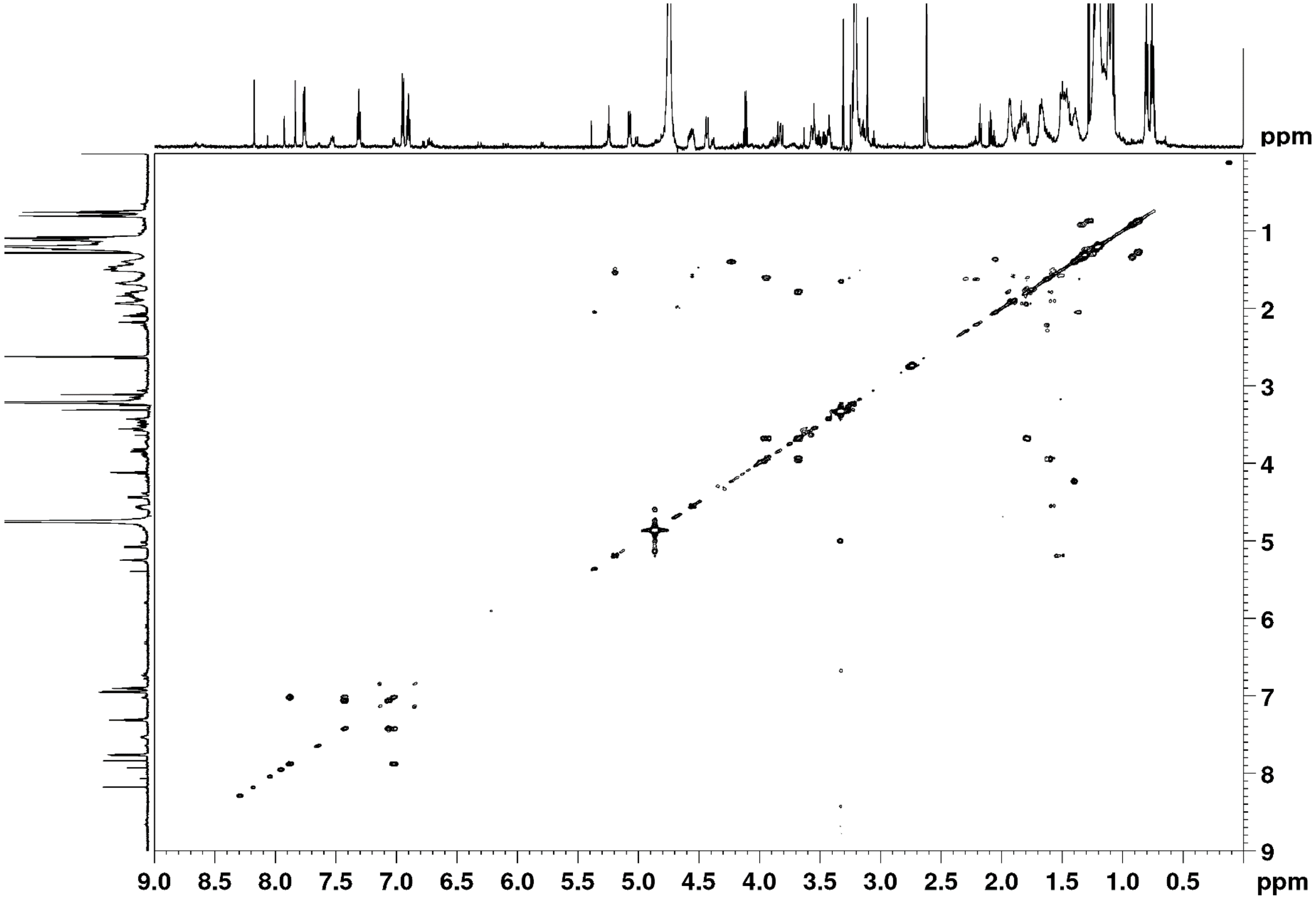

Figure S28: COSY spectrum of **5** (600 MHz, methanol-d_4, 25 °C)

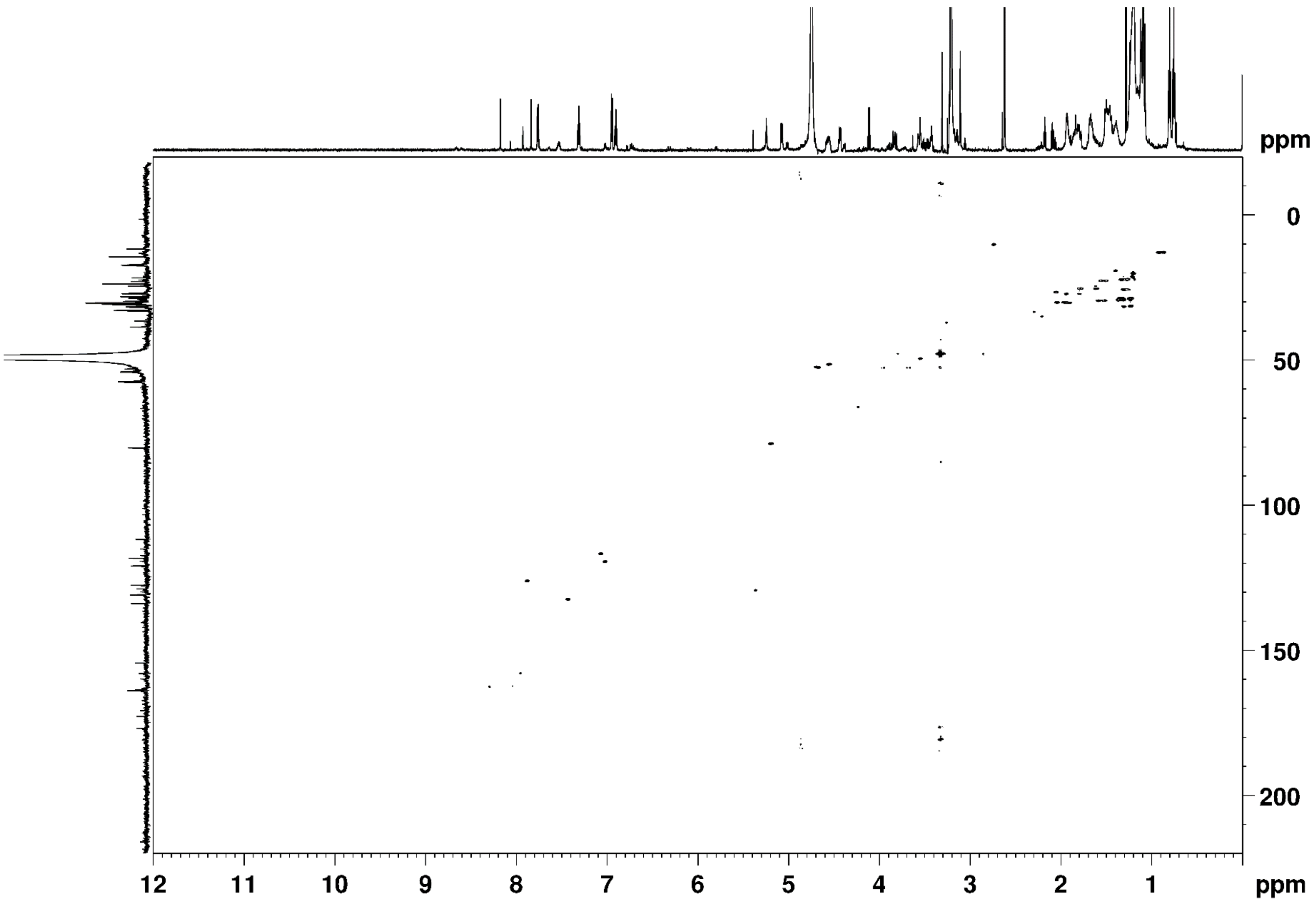

Figure S29: HSQC spectrum of **5** (600 MHz, methanol-d_4, 25 °C)

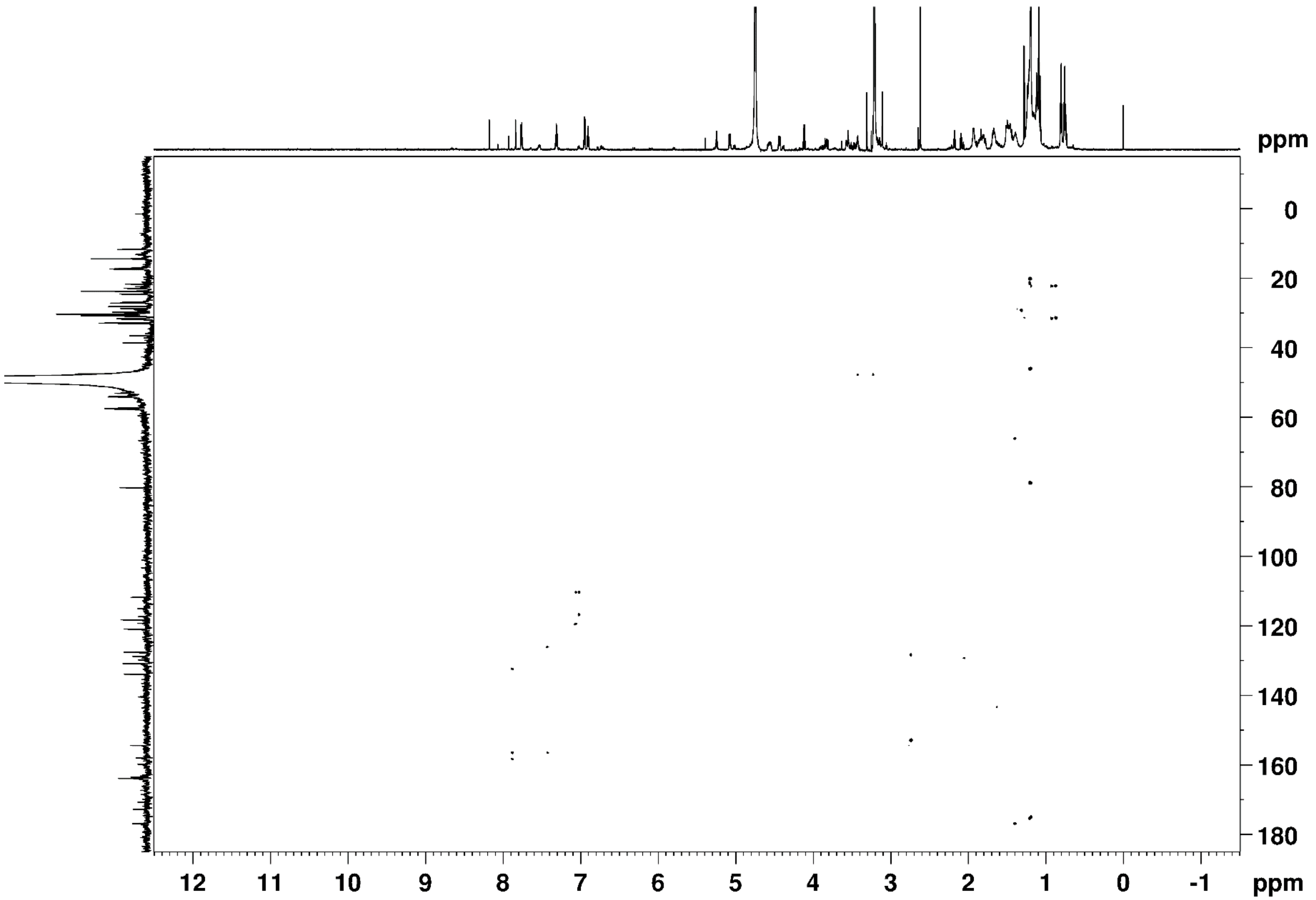

Figure S30: HMBC spectrum of **5** (600 MHz, methanol-d_4, 25 °C)

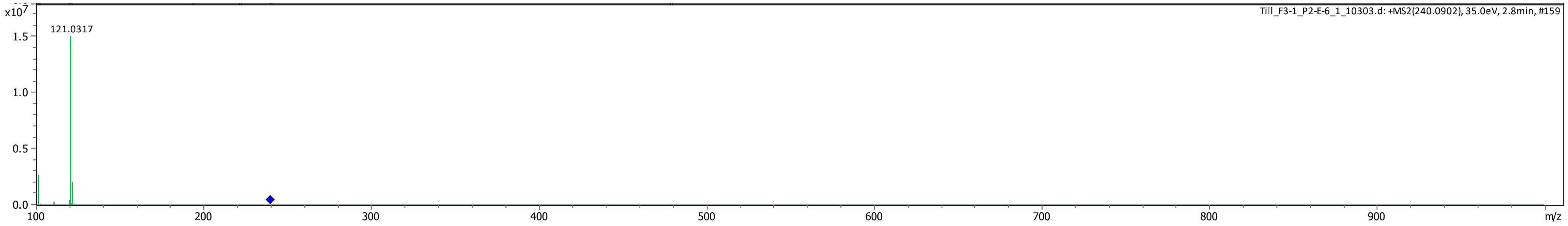

Figure S31: MS/MS spectrum of **6**

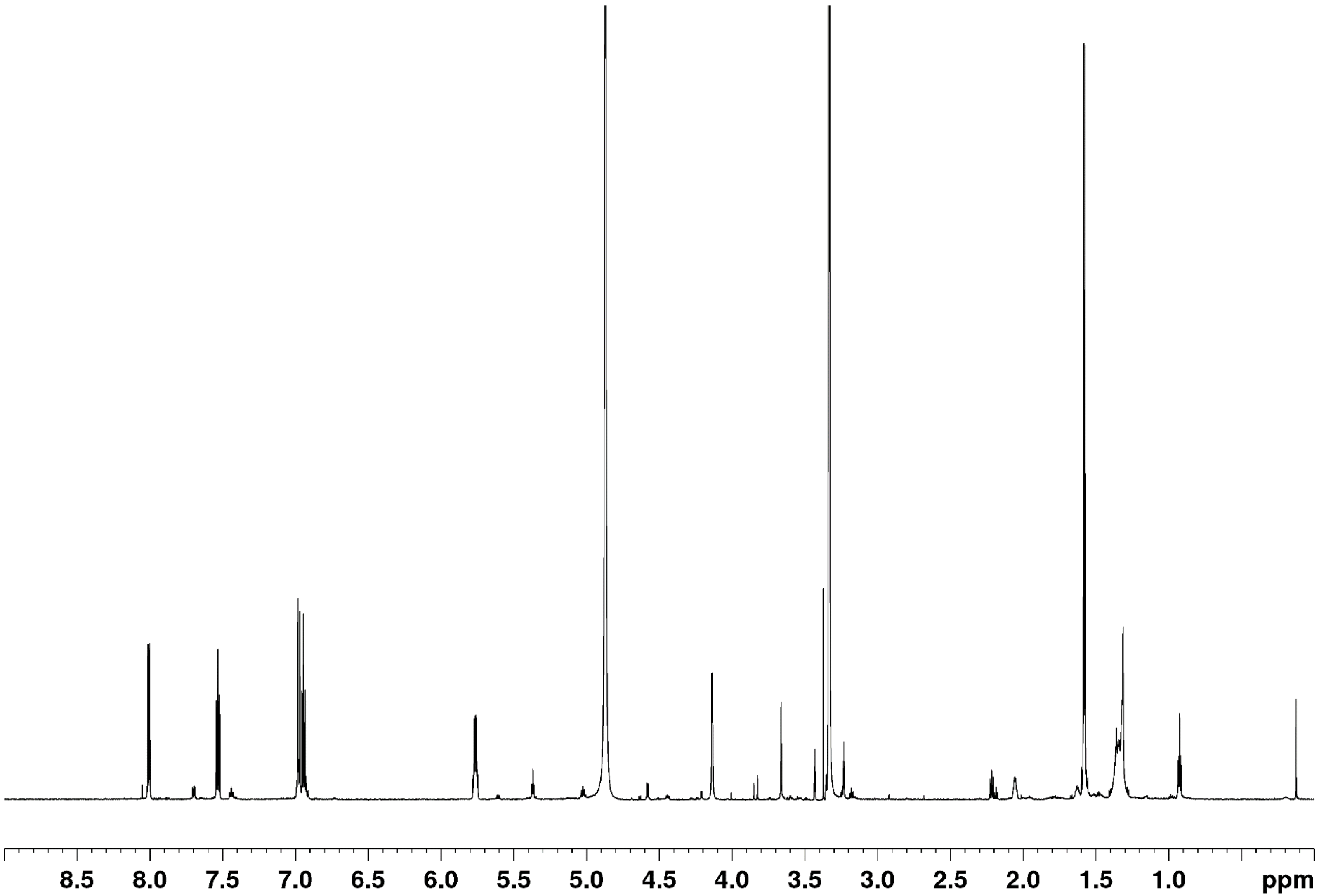

Figure S32: ^{1}H NMR spectrum of **6** (600 MHz, methanol-d_4, 25 °C)

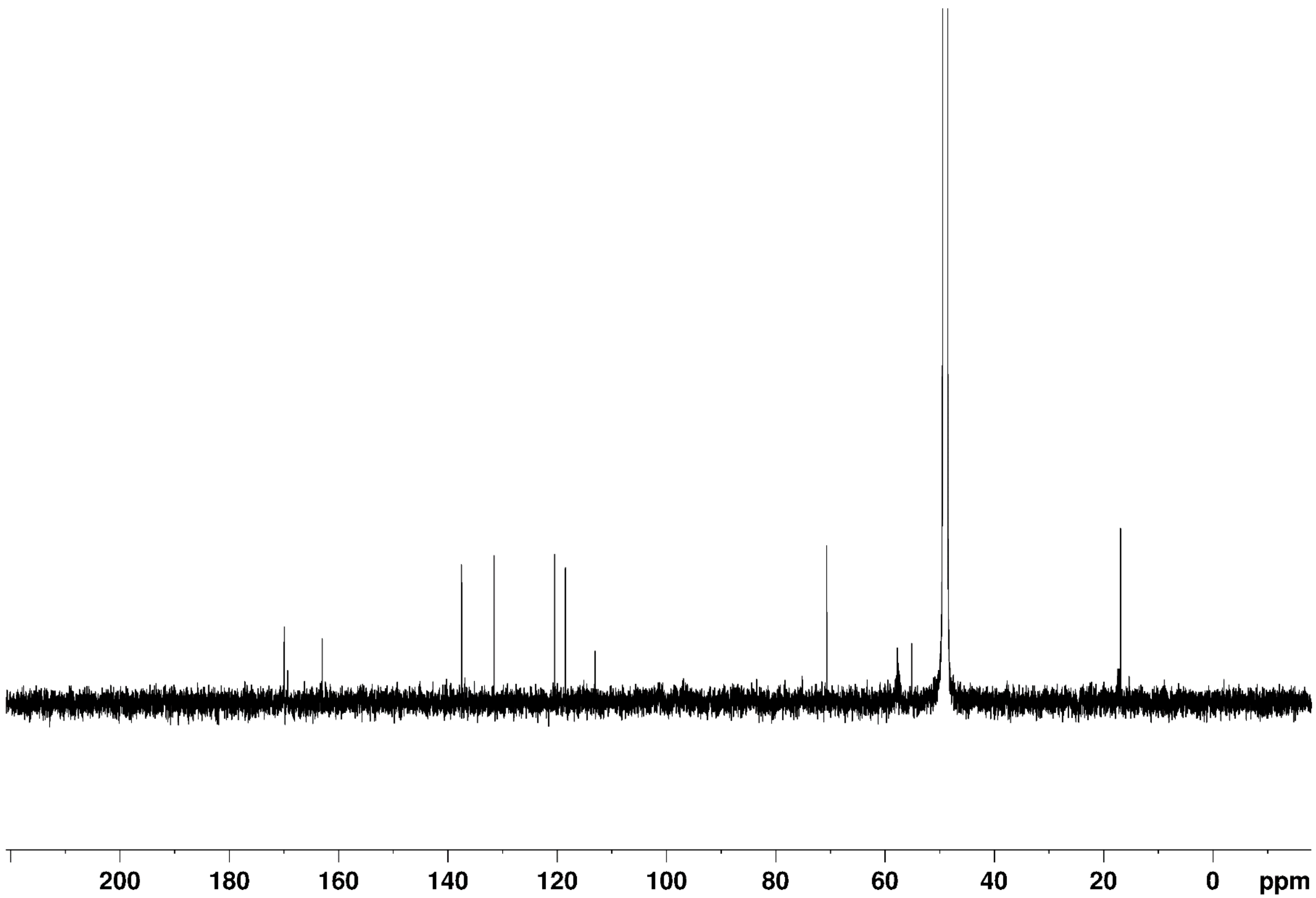

Figure S33: ^{13}C NMR spectrum of **6** (150 MHz, methanol-d_4, 25 °C)

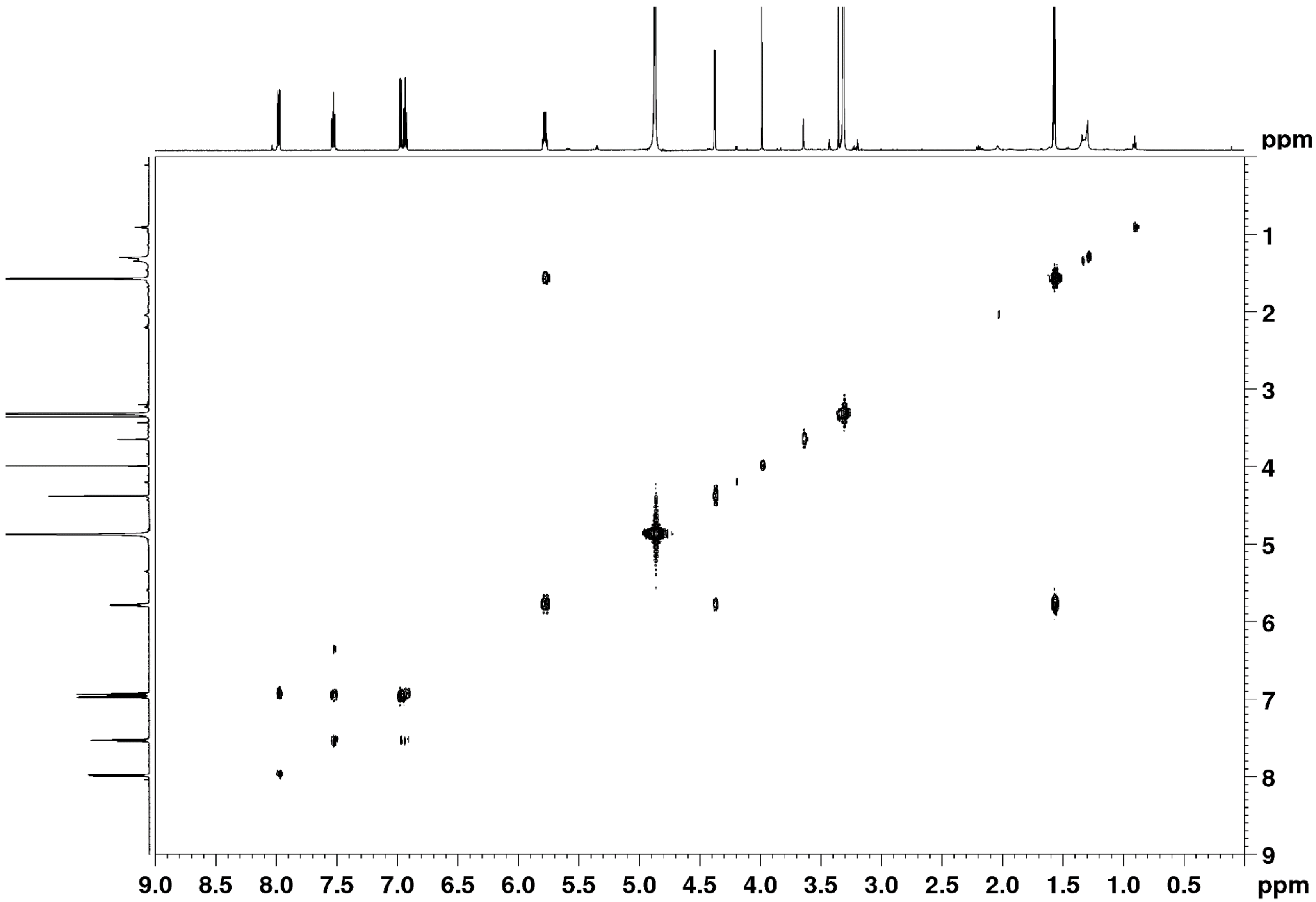

Figure S34: COSY spectrum of **6** (600 MHz, methanol-d_4, 25 °C)

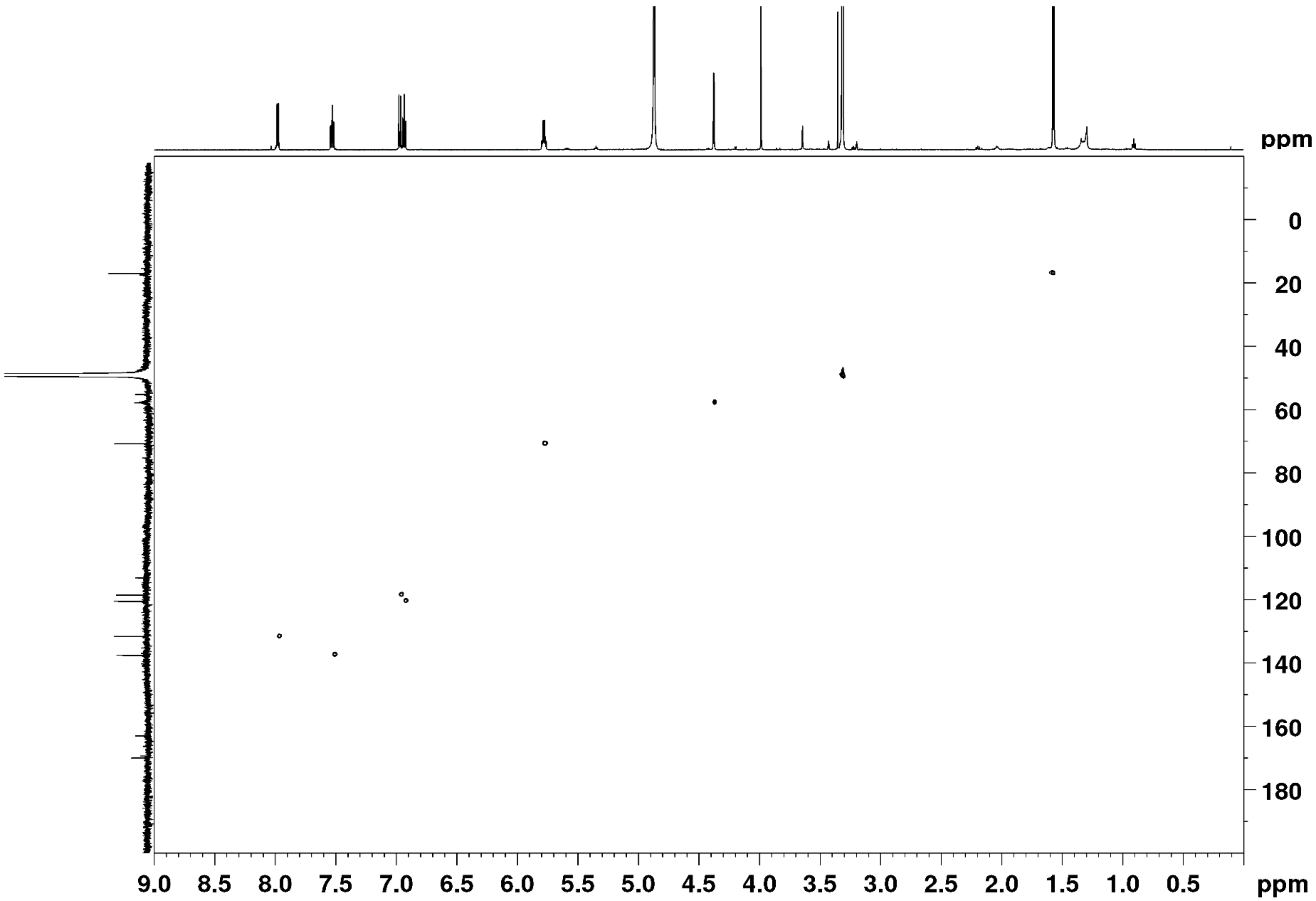

Figure S35: HSQC spectrum of **6** (600 MHz, methanol-d_4, 25 °C)

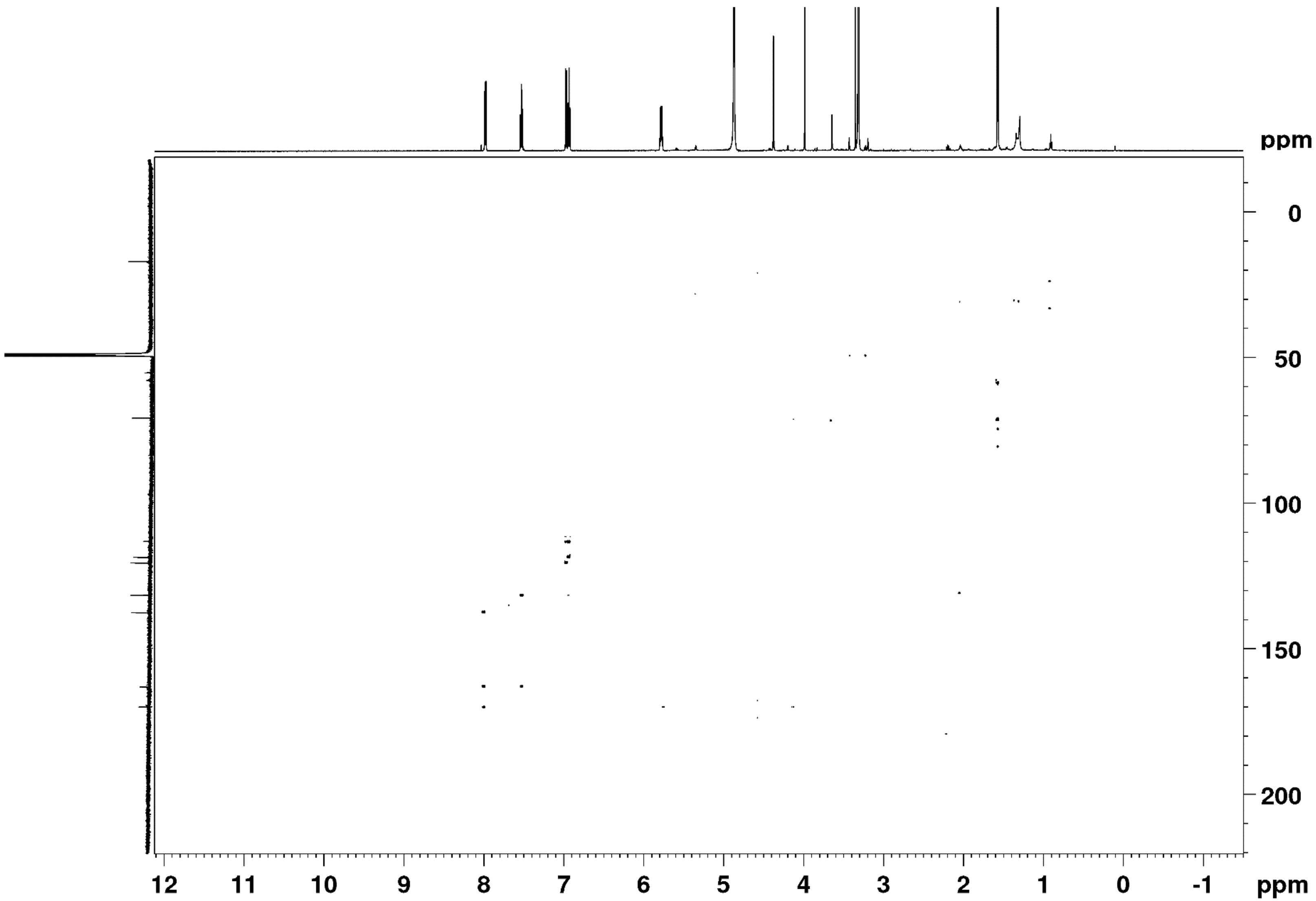

Figure S36: HMBC spectrum of **6** (600 MHz, methanol-d_4, 25 °C)
